# Exome sequencing identifies novel and recurrent mutations in *GJA8* and *CRYGD* associated with inherited cataract

**DOI:** 10.1186/s40246-014-0019-6

**Published:** 2014-11-18

**Authors:** Donna S Mackay, Thomas M Bennett, Susan M Culican, Alan Shiels

**Affiliations:** Department of Ophthalmology and Visual Sciences, Washington University School of Medicine, 660 S. Euclid Ave., Box 8096, St. Louis, Missouri 63110 USA

**Keywords:** Cataract, Exome sequencing, *CRYGD*, *GJA8*

## Abstract

**Background:**

Inherited cataract is a clinically important and genetically heterogeneous cause of visual impairment. Typically, it presents at an early age with or without other ocular/systemic signs and lacks clear phenotype-genotype correlation rendering both clinical classification and molecular diagnosis challenging. Here we have utilized trio-based whole exome sequencing to discover mutations in candidate genes underlying autosomal dominant cataract segregating in three nuclear families.

**Results:**

In family A, we identified a recurrent heterozygous mutation in exon-2 of the gene encoding γD-crystallin (*CRYGD*; c.70C > A, p.Pro24Thr) that co-segregated with ‘coralliform’ lens opacities. Families B and C were found to harbor different novel variants in exon-2 of the gene coding for gap-junction protein α8 (*GJA8*; c.20T > C, p.Leu7Pro and c.293A > C, p.His98Pro). Each novel variant co-segregated with disease and was predicted *in silico* to have damaging effects on protein function.

**Conclusions:**

Exome sequencing facilitates concurrent mutation-profiling of the burgeoning list of candidate genes for inherited cataract, and the results can provide enhanced clinical diagnosis and genetic counseling for affected families.

**Electronic supplementary material:**

The online version of this article (doi:10.1186/s40246-014-0019-6) contains supplementary material, which is available to authorized users.

## Background

Hereditary forms of cataract constitute a clinically and genetically heterogeneous condition affecting the ocular lens [1–3]. Typically, inherited cataract has an early onset (<40 years) and most cases are diagnosed at birth (congenital), during infancy, or during childhood accounting for 10%–25% of all pediatric cataract cases [2]. Congenital and infantile forms of cataract are a clinically important cause of impaired visual development that accounts for 3%–39% of childhood blindness, worldwide [4]. Despite advances in surgical treatment, pediatric cataract poses a long-term risk of postoperative complications including secondary glaucoma, nystagmus, and retinal detachment [5–9].

Cataract can be inherited, either, as an isolated lens phenotype—usually with autosomal dominant transmission and full penetrance—or as part of a genetic/metabolic disorder (http://www.omim.org) involving additional ocular defects (e.g., anterior segment dysgenesis MIM107250) and/or systemic abnormalities (e.g., galactosemia MIM230400). Under slit-lamp examination, inherited cataract exhibits considerable inter- and intrafamilial phenotypic variation in location, size, shape, density, progression rate, and even color of the lens opacities [10]. Currently, genetic studies have identified over 39 genes and loci for inherited cataract, with or without other ocular signs [1,3]. These include gene coding for α-, β-, and γ-crystallins (e.g., *CRYAA*, *CRYBB2*, *CRYGD*), α-connexins (*GJA3*, *GJA8*) and other lens membrane or cytoskeleton proteins (e.g., *MIP*, *BFSP2*), several transcription factors (e.g., *HSF4*, *PITX3*), and an expanding group of functionally divergent genes (e.g., *EPHA2*, *TDRD7*, *FYCO1*). Since mutations in the same gene can cause morphologically different lens opacities and mutations in different genes can cause similar opacities, there is little genotype-phenotype correlation for inherited cataract rendering both clinical classification and molecular diagnosis challenging.

Traditionally, linkage analysis in extended pedigrees has been used to map cataract disease loci to specific chromosome regions and thereby limit the number of positional candidate genes that need to be conventionally sequenced in order to discover underlying mutations. However, the advent of next-generation (massively parallel) sequencing has facilitated the concurrent screening of multiple candidate genes in nuclear families and cases without a family history. Here, we have undertaken affected child-parent-trio-based whole-exome next-generation sequencing in order to identify mutations underlying autosomal dominant cataract in three nuclear families.

## Results

### Cataract families

**Figure 1:**
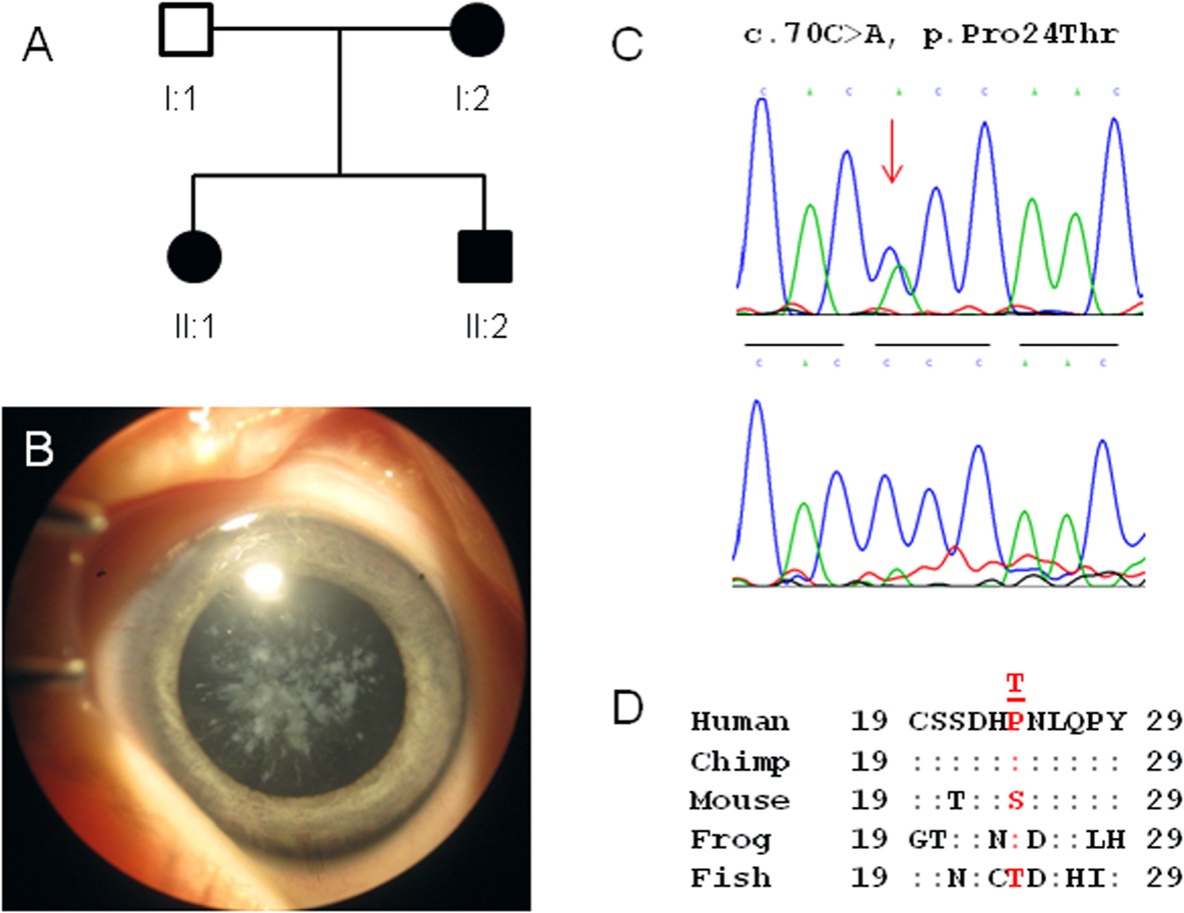
**Mutation analysis of inherited cataract in family A. (A)** Pedigree of family A. Squares denote males, circles denote females, and filled symbols denote affected status. The trio of individuals I:1, I:2, and II:1 was subject to exome sequencing. **(B)** Photograph of coralliform lens opacities in the left eye of individual II:2 just prior to surgery at 3 months of age. **(C)** Sanger sequence of *CRYGD* showing the heterozygous c.70 C > A and p.Pro24Thr mutation found in affected individuals I:2, II:1, and II:2 (upper trace) but not in the unaffected spouse I:1 (lower trace). Horizontal bars indicate the codon reading frame. **(D)** Amino acid alignment of *CRYGD* showing low cross-species conservation of Pro24.

**Figure 2:**
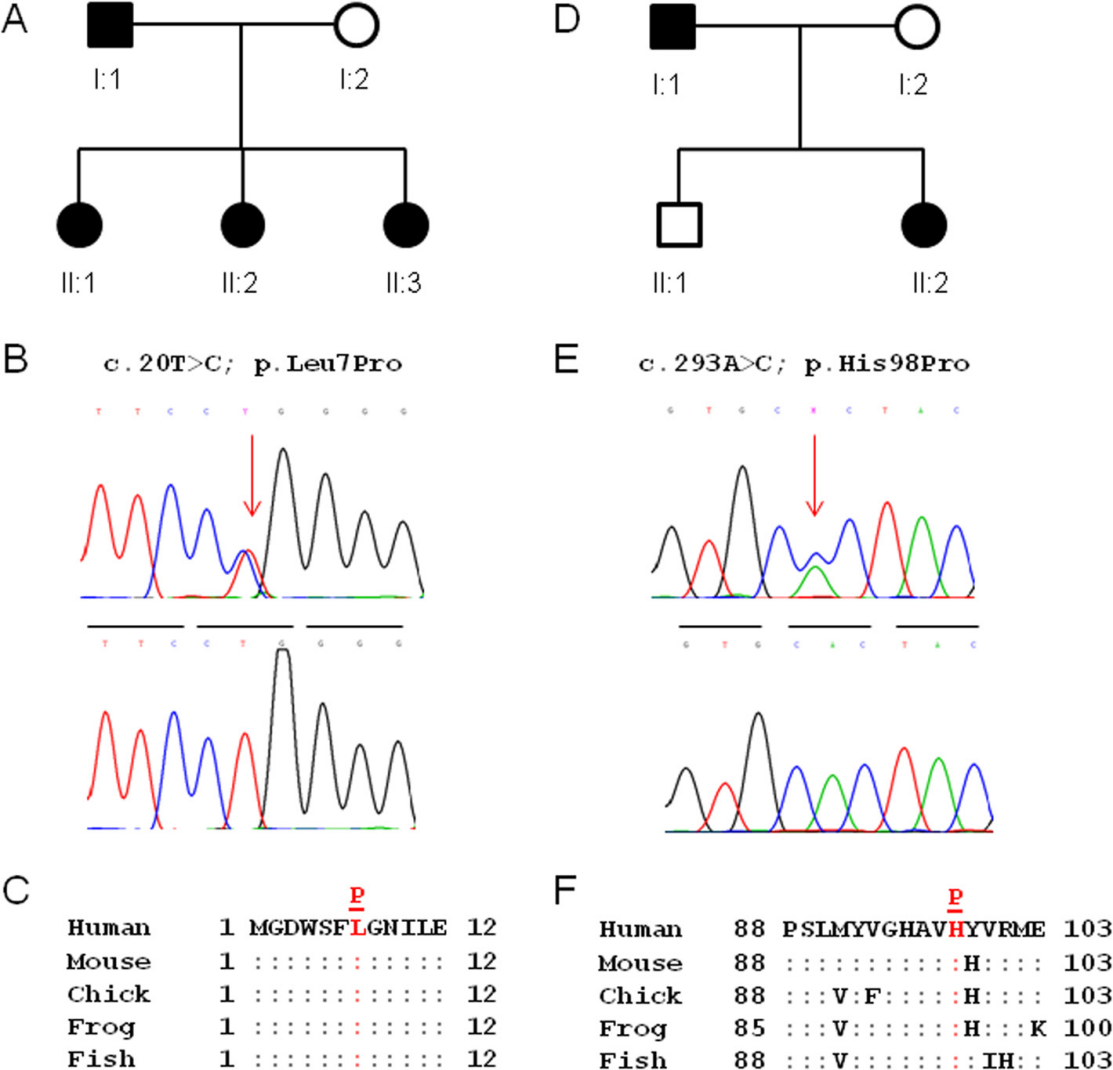
**Mutation analysis of inherited cataract in family B and family C. (A)** Pedigree of family B. The trio of individuals I:1, I:2, and II:1 was subject to exome sequencing. **(B)** Sanger sequence of *GJA8* showing the heterozygous c.20 T > C, and p.Leu7Pro mutation found in affected individuals I:1, II:1, II:2, and II:3 but not in the affected spouse I:2 (lower trace). Horizontal bars indicate the codon reading frame. **(C)** Amino acid alignment of *GJA8* showing high cross-species conservation of Leu7. **(D)** Pedigree showing family C. The trio of individuals I:1, I:2, and II:2 was subject to exome sequencing. **(E)** Sanger sequence of *GJA8* showing the heterozygous c. 293A > C and p.His98Pro mutation found in affected individuals I:1 and II:2 (upper trace) but not in the unaffected individuals I:2 and II:1 (lower trace). Horizontal bars indicate the codon reading-frame. **(F)** Amino acid alignment of *GJA8* showing high cross-species conservation of His98.

We investigated three Caucasian-American pedigrees segregating cataract with autosomal dominant transmission in the absence of other ocular and/or systemic abnormalities (Figures 1A and 2A,D). A review of ophthalmic records indicated that bilateral cataract was diagnosed at birth (congenital) or during infancy in all three families with age-at-surgery ranging from 3 months to 1 year. In family A, the lens opacities appeared similar to those first described by Gunn in 1895 as resembling a piece of coral or coralliform [11]. No clinical images of lens opacities were available for family B or C, and none of the families had a sufficient number of meiotic events (≥10) to support independent linkage analysis. Instead, an affected child–parent plus spouse trio from each family was selected for whole-exome sequencing.

### Candidate genes and exome sequences

We pre-selected 39 candidate genes for inherited cataract (Additional file 1) cited in the OMIM (http://www.omim.org), Cat-Map (http://cat-map.wustl.edu/), and iSyTE (http://bioinformatics.udel.edu/Research/iSyTE) databases [3,12]. The candidate list comprises genes known to be highly expressed in the lens including those coding for cystallins, connexins, and other lens membrane/cytoskeletal proteins, along with several more widely expressed genes that are associated with cataract and other limited eye/systemic conditions. Collectively, these candidate genes span over 111,000 bps of the genome and contain 300 exons located on chromosomes 1–13, 16, 17, 19–22, and X.

For all nine exome samples, over 98% of total paired-end reads were mapped to the reference genome (Additional file 2). Approximately 72%–84% of mapped reads were present in the captured exomes, and the average mean-mapped read-depth was 149.2X. With the exception of one sample in family C (C-I:1), >97% of each exome achieved a read-depth of ≥10X coverage, yielding a total of >38,900 single nucleotide polymorphisms (SNPs), of which >8,400 were non-synonymous and >1,400 were novel. For exome C-I:1, 80.61% reached ≥10X coverage yielding a total of 34,435 SNPs (7,639 non-synonymous and 1,331 novel). In addition, exome C-I:1 contained several more unexpected regions of low coverage (gaps) than those detected in the other exomes (Additional file 2). However, the reduced coverage of exome C-I:1 did not compromise analysis of variants in the candidate genes of interest. Coverage of the 39 candidate genes exceeded a read-depth of >10X with three exceptions. The iron response element (IRE) of *FTL* is located in the 5′-UTR (untranslated region) and was not covered by capture probes. In addition, coverage of the single exons that code for *FOXE3* and *MAF* was incomplete as previously reported [13]. We excluded mutations in all three missing gene regions by Sanger sequencing of an affected member of each family essentially as described [13,14]. Collectively, from the nine exomes sequenced, 112 variants were identified in 32 of the 39 candidate genes (Additional file 3). Of these variants, only five did not have genome reference sequence (rs) numbers and were potentially novel variants.

### Family A variants

A review of the exome SNPs in family A with the list of candidate genes for cataract identified a total of 76 variants in 28 of 39 genes (Additional file 1 and Additional file 3). Of these, six variants (two coding/missense and four non-coding/synonymous) in five candidate genes were found in both affected relatives and not in the unaffected spouse. However, five of these variants associated with four candidate genes (*SLC16A12*, *PAX6*, *CRYAB*, *GALK1*) were excluded as disease-causing mutations as they have minor allele frequencies (MAFs) >0.01% (range 8.5%–52.4%) in Caucasians (Additional file 3). We note that the variant rs3740030 in *SLC16A12* on chromosome 10 (chr10:91,222,287) has previously been associated with age-related cataract [15]. As rs3740030 was first thought to be a non-coding variant located in the 5′-UTR, the authors proposed a complex functional mechanism that involved modulation of translational efficiency. However, rs3740030 is now known to be located in exon-3 of *SLC16A12* (c.49T > G) and was predicted to result in a non-conservative tryptophan-to-glycine substitution at codon 17 (p.Trp17Gly). While this variant was also predicted *in silico* to have a damaging effect on protein function (PolyPhen-2 score =0.997), it had a MAF value of 8.5% in Caucasians, suggesting that it is unlikely to be disease causing in family A. The remaining variant, rs28931605, occurred in exon-2 of *CRYGD* (c.70C > A) on chromosome 2 (chr2:208,989,018) and was predicted to result in the non-conservative substitution of proline-to-threonine at codon 24 (p.Pro24Thr) (Table 1). While this variant was predicted *in silico* to be tolerated, benign, or neutral with respect to protein function (Table 2), it has been previously associated with autosomal dominant cataract in multiple families (Additional file 4). The p.Pro24Thr variant has also been documented as p.Pro23Thr based on N-terminal processing of the of the *CRYGD* protein that removes the initiator methionine residue. Here, we have adopted the recommended nomenclature in order to avoid confusion and re-numbering of other mutations in *CRYGD* associated with inherited cataract [16]. Sanger sequencing of all four members of family A (Figure 1A,C) confirmed that the p.Pro24Thr variant co-segregated with disease providing further support for its role as a causal mutation.

**Table 1:** **Summary of mutations detected by exome sequencing of trios from families A, B, and C**

**Pedigree (numbers affected)**	**Physical location of variant**	**Candidate gene (ID)**	**Exon**	**cDNA variant**	**Protein variant**	Allele frequency (EVS)*	**Status**
A (3)	chr2:208,989,018	*CRYGD* (1412)	2	c.70C > A	p.Pro24Thr	0/8,600	Recurrent (Additional file 5)
B (4)	chr1:147,380,102	*GJA8* (2703)	2	c.20T > C	p.Leu7Pro	0/8,600	Novel
C (2)	chr1:147,380,375	*GJA8* (2703)	2	c.293A > C	p.His98Pro	0/8,600	Novel

*Allele frequencies for European Americans listed on the Exome Variant Server.

**Table 2:** ***In silico*** **predictions of functional effects for the three mutations identified in this study**

**Prediction program**		***CRYGD***	***GJA8***	***GJA8***
		**c.70C > A**	**c.20T > C**	**c.293A > C**
		**p.Pro24Thr**	**p.Leu7Pro**	**p.His98Pro**
SIFT	Value (<0.05)	0.10	0.00	0.00
	Prediction	Tolerated	Not tolerated	Not tolerated
Polyphen-2	Score	0.084	0.991	1.000
	Prediction	Benign	Probably damaging	Probably damaging
PMUT	NN output	0.0936	0.8749	0.7822
	Reliability	8	7	5
	Prediction	Neutral	Pathological	Pathological
PANTHER	subPSEC (<−3)	−2.35974	−4.3388	−3.98807
	P_deleterious_ (>0.5)	0.34519	0.79148	0.72871
PON-P2	Probability for pathogenicity	0.479	0.947	0.444
	Standard error	0.332	0.050	0.090
	Prediction	Unknown	Pathogenic	Unknown
MutPred	Probability of deleterious mutation	0.840	0.918	0.889
	Molecular mechanisms disrupted		Loss of stability (*p* =0.0067)	
			Gain of disorder (*p* =0.0099)	

### Family B variants

A review of the exome SNPs in family B with the candidate gene list, revealed a total of 73 variants in 22 of 39 genes (Additional file 1 and Additional file 3). Only 13 of these variants (12 non-coding or synonymous) associated with 7 of the candidate genes were found in both affected relatives and not in the unaffected spouse. All 12 non-coding or synonymous variants had MAF values >0.01% (range 0.4%–45.80%) and were effectively excluded as disease-causing mutations. The remaining variant was located in exon-2 of *GJA8* (c.20T > C) on chromosome 1 (chr1:147,380,102) and was predicted to result in the substitution of leucine-to-proline at codon 7 (p.Leu7Pro) (Table 1). The p.Leu7Pro substitution represented a relatively conservative change with the non-polar, side-chain of leucine ([CH_3_]_2_-CH-CH2-) replaced by the unusual non-polar, side-ring of proline (−CH_2_-CH_2_-CH_2_-). However, Leu7 is phylogenetically conserved in *GJA8* (Figure 2C), and the Pro7 substitution was predicted *in silico* to have probably damaging effects on protein function (Table 2). Sanger sequencing of all five members of family B (Figure 2A,B) confirmed that the novel p.Leu7Pro variant in *GJA8* co-segregated with cataract further suggesting that it was the disease-causing mutation.

### Family C variants

A review of the exome SNPs in family C using the candidate gene list yielded a total of 82 variants in 23 of the 39 genes (Additional file 1 and Additional file 3). However, only three of these variants associated with the candidate genes, *WFS1*, *BFSP1*, and *GJA8*, were present in both affected relatives but not in the unaffected spouse. The variants associated with *WFS1* (rs734312) and *BFSP1* (rs2281207) had MAF values of 54.69% and 25.74%, respectively, and were excluded as causative mutations. The remaining variant occurred in exon-2 of *GJA8* (c.293A > C) on chromosome 1 (chr1:147,380,375) and was predicted to result in a non-conservative substitution of histidine-to-proline at codon 98 (p.His98Pro) (Table 1). Histidine 98 is phylogenetically conserved across species (Figure 2F), and this variant was also predicted to have damaging effects on protein function using six mutation prediction programs (Table 2). Sanger sequencing of all four members of family C (Figure 2D,E) confirmed that the novel p.His98Pro variant in *GJA8* co-segregated with cataract, consistent with it being the disease-causing mutation.

## Discussion

Several recent studies have employed exome sequencing of index patients or probands in multiple families in order to discover mutations in candidate genes underlying autosomal dominant and recessive forms of cataract [13,17–19]. In this study, we have used trio-based exome sequencing to uncover a recurrent missense mutation in *CRYGD* (p.Pro24Thr) and two novel missense mutations in *GJA8* (p.Leu7Pro, p.His98Pro) associated with autosomal dominant cataract in three nuclear families. Child–parent trios offer the initial benefit of co-segregation testing during exome variant analysis, but this advantage may be offset in larger cohorts of families by the additional sequencing costs. The p.Pro24Thr substitution in *CRYGD* has now been identified in some 14 different families, mostly segregating coralliform cataract that affects more than 133 individuals with varied ethnic backgrounds and constitutes the most recurrent missense mutation in a crystallin gene to be associated with inherited cataract (Additional file 4). The novel mutations found in *GJA8* increase the mutation spectrum of this connexin gene to at least 32 different mutations segregating in 38 families making it one of the most common non-crystallin genes to be associated with inherited cataract in humans (Additional file 5).

*CRYGD* (MIM: 123690) consists of three exons and encodes γD-crystallin—a hydrophylic protein of 174 amino acids that is characterized by two βγ-crystallin domains each formed by two repeat Greek-key motifs of approximately 40 residues. *CRYGD* is expressed at high concentrations in fiber cells of mammalian lenses and plays an important structural role in establishing lens transparency and gradient refractive index [20]. Proline at position 24 is located within the first Greek-key motif of human *CRYGD* but is not well conserved across species (replaced by serine in the mouse and threonine in the zebrafish). Consequently, *in silico* analysis predicted that the Pro24Thr substitution was benign (Table 2). Further, NMR-spectroscopy and X-ray crystallography have indicated that the Pro24 and Thr24 proteins are structurally similar overall [21,22]. However, the Thr24 mutant exhibits local conformational and dynamic differences that may initiate aggregation or polymerization and *in vitro* experiments have shown that the Thr24 protein exhibits reduced solubility—a property that is likely to trigger cataract formation [23–25].

*GJA8* (MIM: 600897) comprises two exons with exon-2 coding for the entire 433 amino acid residues of gap-junction protein α8 or connexin 50. *GJA8* contains four transmembrane domains that are joined by two extracellular loops and one cytoplasmic loop and flanked by cytoplasmic N- and C-termini. By forming hexamers, or hemi-channels, that can dock between adjacent cells to create gap-junction channels, *GJA8* plays an important role in lens intercellular communication [26]. Of the 32 known coding mutations in *GJA8*, 30 result in missense substitutions that, with one exception, are associated with autosomal dominant cataract, and the remaining two are frameshift mutations associated with autosomal recessive cataract (Additional file 5). Most of the missense substitutions are located within the N-terminal half of the protein, which also contains the conserved connexin domain (pfam00029; amino acids 3–109). The novel p.Leu7Pro substitution found in family B is the first to be located at the cytosolic N-terminal end of human *GJA8*. Support for its pathogenicity in humans is provided by the SHR-Dca rat strain, which inherits semi-dominant cataract [27]. Heterozygous (+/Dca) mutants develop nuclear pulverulent opacities and smaller eyes than wild-type, while homozygotes (Dca/Dca) present with severe microphthalmia and a hypoplastic lens. The underlying mutation has been identified as a missense mutation in *GJA8* (c.20T > A) that is predicted to result in a non-conservative p.Leu7Gln substitution. Both the rat p.Leu7Gln and human p.Leu7Pro mutations result in the substitution of a highly conserved leucine residue with uncharged amino acids, suggesting that they may exert similar deleterious effects on *GJA8* function.

The novel p.His98Pro mutation identified in family C, is located near the junction of the second transmembrane domain with the cytoplasmic loop of *GJA8*. Four other mutations, p.Val79Leu, p.Pro88Ser, p.Pro88Gln, and p.Pro88Thr, have previously been localized to the second transmembrane domain (Additional file 5). Functional expression studies of the relatively conservative p.Val79Leu substitution results in functional gap-junction channels with altered voltage-gating and a reduction in the single-channel open probability [28]. By contrast, neither of the non-conservative p.Pro88Gln and p.Pro88Ser substitutions was targeted to the plasma membrane, with the former accumulating in the endoplasmic-reticulum(ER)-Golgi-complex and the latter forming discrete cytoplasmic inclusions [26]. Based on the non-conservative nature of the p.His98Pro substitution, we speculate that this mutant will also fail to reach the plasma membrane and form functional gap-junction channels

## Conclusions

Exome sequencing provides a rational approach to concurrently screen over 39 candidate genes for inherited cataract in nuclear families or even sporadic cases. In addition, exome sequencing may enable the discovery of novel genes underlying inherited cataract and, potentially, genes associated with age-related cataract. However, considerable supporting evidence (e.g., additional mutations, functional expression *in vitro*, and/or an animal model) will be required to verify disease causation. In a clinical setting, results from exome sequencing are unlikely to be ‘clinically actionable’ with respect to surgical treatment and subsequent management of inherited cataract. However, such data can contribute to a gene-centric clinical classification of inherited cataract and provide enhanced diagnosis and genetic counseling for affected families.

## Methods

### Ethics statement

Ethical approval for this study was obtained from the Washington University Human Research Protection Office (HRPO), and written informed consent was provided by all participants prior to enrollment in accordance with the tenets of the Declaration of Helsinki and Health Insurance Portability and Accountability Act (HIPAA) regulations.

### Family participants

Three Caucasian-American pedigrees segregating autosomal dominant cataract were ascertained through ophthalmic records in the Department of Ophthalmology and Visual Sciences at Washington University School of Medicine. Blood samples were obtained from available family members including a spouse (Figures 1 and 2). Leukocyte genomic DNA was purified using the Gentra Puregene Blood kit (Qiagen, Valencia, CA) and quantified by absorbance at 260 nm (NanoDrop 2000, Wilmington, DE).

### Exome sequencing

Whole exome capture was achieved using the SureSelect Human All Exon V5 (50.4 Mb) Kit, according to manufacturer’s instructions (Agilent Technologies). Briefly, genomic DNA (3 μg) was fragmented (150–200 bp) by acoustic shearing, ligated to adapter primers, and PCR-amplified. Following denaturation (95°C, 5 min), amplified DNA-fragment libraries (~500 ng) were hybridized in a solution under high stringency (65°C, 24 h) with biotinylated RNA capture probes (~120 bp). Resulting DNA/RNA hybrids were recovered by streptavidin-coated magnetic bead separation (Dynal, Invitrogen, Carlsbad, CA). Captured DNA was eluted (NaOH) and then subject to solid phase (flow-cell) next-generation (massively parallel) sequencing on a HiSeq2000 System (Illumina, San Diego, CA) using the Illumina Multiplexing Sample Preparation Oligo-nucleotide Kit and the HiSeq 2000 Paired-End Cluster Generation Kit according to the manufacturer’s instructions. Briefly, hybrid-capture libraries were amplified to add indexing (identifying) tags and sequencing primers then subjected to paired-end (2 × 101 bp read length), multiplex sequencing-by-synthesis using fluorescent, cyclic reversible (3′-blocked) terminators. A pool of three exome samples (representing a family trio) was sequenced in a single lane of the sequencer’s flow-cell.

### Exome variant analysis

Raw sequence data was aligned to the human reference genome (build hg19) by NovoalignMPI (www.novocraft.com), and sequence variants called using the Sequence Alignment/Map format (SAMtools) and Picard programs (http://samtools.sourceforge.net/) and further annotated using SeattleSeq (http://snp.gs.washington.edu/SeattleSeqAnnotation138/). Target coverage and read-depth were reviewed by the Integrated Genomics Viewer (IGV; http://www.broadinstitute.org/igv/). Variants were filtered using the Ingenuity variant analysis website (IVA http://ingenuity.com) or the gNOME project pipeline (http://gnome.tchlab.org/) [29]. Identified variants in the pre-selected candidate genes (Additional file 1) were then reviewed for presence/absence and frequency in various websites including dbSNP (http://www.ncbi.nlm.nih.gov/snp/), 1000 genomes (http://www.1000genomes.org/), and the Exome Variant Server database (http://evs.gs.washington.edu/EVS/). The predicted effect on protein function was analyzed using the SIFT (http://sift.jcvi.org), PolyPhen-2 (http://genetics.bwh.harvard.edu/pph2/), PMUT (http://mmb2.pcb.ub.es:8080/PMut/), PON-P2 (http://structure.bmc.lu.se/PON-P2/), PANTHER (http://www.pantherdb.org/tools/csnpScoreForm.jsp), and MutPred (http://mutpred.mutdb.org/) *in silico* mutation prediction programs [30–34].

### Sanger sequencing

Genomic DNA (2.5 ng/μl, 10 μl reactions) was amplified (35 cycles) in a GeneAmp 9700 thermal cycler using Top Taq mastermix kit (Qiagen) and 20 pmol of gene-specific primers (Additional file 6). Resulting PCR amplicons were enzyme-purified with ExoSAP-IT (USB Corporation, Cleveland, OH). The purified amplicons were direct cycle-sequenced in both directions with BigDye Terminator Ready Reaction Mix (v3.1)(Applied Biosystems, Grand Island, NY) containing M13 forward or reverse sequencing primers, then ethanol precipitated and detected by capillary electrophoresis on a 3130xl Genetic Analyzer running Sequence Analysis (v.6.0) software (Applied Biosystems) and Chromas (v2.23) software (Technelysium, Tewantin, Queensland, Australia).

## Additional files

Additional file 1: Table S1. Candidate genes for inherited cataract evaluated in this study. Additional file 2: Table S2. Sample metrics for exome sequencing of trios from families A, B, and C. Additional file 3: Table S3. Exome sequencing variants found in candidate genes for inherited cataract (Additional file 1, Table S1) in family trios A, B, and C. Additional file 4: Table S4. Recurrence of the p.Pro24Thr mutation in *CRYGD* associated with autosomal dominant cataract. Additional file 5: Table S5. Mutation profile of *GJA8*. Additional file 6: Table S6. PCR primers used for Sanger sequencing of mutations found in *CRYGD* and *GJA8*. Each primer used was tailed with M13 sequences to aid in Sanger sequencing. The forward primers were tagged with ‘tgtaaaacgacggccagt’ and the reverse primers with ‘caggaaacagctatgacc’.

## Abbreviations

OMIM: Online Mendelian Inheritance in Man
MAF: Minor allele frequency
SHR-Dca: Spontaneously hypertensive rat-Dominant cataract
NMR: Nuclear magnetic resonance
