# Supplementary material for: Exome sequencing identifies novel and recurrent mutations in GJA8 and CRYGD associated with inherited cataract
Source: Hum Genomics. 2014 Nov 18;8(1):19. doi: 10.1186/s40246-014-0019-6 (PMC4240822; doi:10.1186/s40246-014-0019-6)
Supplement: Additional file 1: Table S1. — Candidate genes for inherited cataract evaluated in this study. [file 40246_2014_19_MOESM1_ESM.docx]

**Additional file 1. Table S1.** Candidate genes for inherited cataract evaluated in this study.

|  | **Locus** | **Gene** | **Name** | **Gene ID** | **MIM ID** | **Inheritance** | **Associated phenotypes** |
| --- | --- | --- | --- | --- | --- | --- | --- |
| **1** | 1p36.13 | *EPHA2* | EPH receptor A2 | 1969 | 176946 | AD/AR |  |
| **2** | 1p32 | *FOXE3* | Forkhead box E3 | 2301 | 601094 | AD | Aphakia, Anterior segment mesenchymal dysgenesis |
| **3** | 1q21.1 | *GJA8* | Gap-junction protein alpha 8 | 2703 | 600897 | AD/AR |  |
| **4** | 2p25.3 | *PXDN* | Peroxidasin homolog | 7837 | 605158 | AR | Anterior segment dysgenesis |
| **5** | 2q34 | *CRYGB* | Gamma B crystallin | 1419 | 123670 | AD |  |
| **6** | 2q34 | *CRYBA2* | Beta A2 crystallin | 2395 | 600836 | AD |  |
| **7** | 2q33.3 | *CRYGC* | Gamma C crystallin | 1420 | 123680 | AD | Microcornea |
| **8** | 2q33.3 | *CRYGD* | Gamma D crystallin | 1421 | 123690 | AD |  |
| **9** | 3p21.31 | *FYCO1* | FYVE and coiled-coil domain containing 1 | 79443 | 607182 | AR |  |
| **10** | 3q22.1 | *BFSP2* | Beaded filament structural protein 2 | 8419 | 603212 | AD |  |
| **11** | 3q27.3 | *CRYGS* | Gamma S crystallin | 1427 | 123730 | AD |  |
| **12** | 4p16.1 | *WFS1* | Wolfram syndrome 1 | 7466 | 606201 | AD | Wolfram syndrome, Wolfram-like syndrome, autosomal dominant deafness |
| **13** | 5q31 | *SIL1* | SIL1 nucleotide exchange factor | 64374 | 608005 | AD/AR | Marinesco-Sjogren syndrome |
| **14** | 6p24 | *GCNT2* | Glucosaminyl (N-acetyl) transferase 2, I-branching enzyme | 2651 | 600429 | AR | Adult i blood group |
| **15** | 7q34 | *AGK* | Acylglycerol kinase | 55750 | 610345 | AR | Senger’s syndrome |
| **16** | 8q13.3 | *EYA1* | EYA transcriptional coactivator and phosphatase 1 | 2138 | 601653 | AD | Branchiootic syndrome, Branchiootorenal dysplasia syndrome, anterior segment anomalies |
| **17** | 9q22.33 | *TDRD7* | Tudor domain containing 7 | 23424 | 611258 | AR |  |
| **18** | 10p13 | *VIM* | Vimentin | 7431 | 193060 | AD |  |
| **19** | 10q23.31 | *SLC16A12* | Solute carrier family 16, member 12 | 387700 | 611910 | AD | Microcornea and glucosuria |
| **20** | 10q24.32 | *PITX3* | Paired-like homeodomain transcription factor 3 | 5309 | 602669 | AD | Anterior segment mesenchymal dysgenesis |
| **21** | 11p13 | *PAX6* | Paired box 6 | 5080 | 607108 | AD | Aniridia and Peters anomaly |
| **22** | 11q22.3 | *CRYAB* | Alpha-B crystallin | 1410 | 123590 | AD/AR | Myofibrillar myopathy, Fatal infantile hypertonic myofibrillar myopathy, Dilated cardiomyopathy |
| **23** | 12q13 | *MIP* | Major intrinsic protein | 4284 | 154050 | AD |  |
| **24** | 13q12.1 | *GJA3* | Gap-junction protein alpha 3 | 121015 | 121015 | AD |  |
| **25** | 16q21 | *HSF4* | Heat shock factor 4 | 3299 | 602438 | AD/AR |  |
| **26** | 16q22-q23 | *MAF* | v-maf avian musculoaponeurotic fibrosacrcoma oncogene homolog | 4094 | 177075 | AD | Microcornea |
| **27** | 17q11.2 | *CRYBA1* | Beta A1 crystallin | 1411 | 123610 | AD |  |
| **28** | 17q12 | *UNC45B* | Unc045 homolog B | 146862 | 611220 | AD |  |
| **29** | 17q25.1 | *GALK1* | Galactokinase | 2584 | 604313 | AR | Galactokinase deficiency |
| **30** | 19q13.3-q4 | *FTL*(IRE) | Ferritin light chain (iron response element) | 2512 | 134790 | AD | Hereditary hyperferritinemia-cataract syndrome |
| **31** | 19q13.4 | *LIM2* | Lens intrinsic membrane protein 2 | 3982 | 154045 | AR |  |
| **32** | 20p12.1 | *BFSP1* | Beaded filament structural protein 1 | 631 | 603307 | AR |  |
| **33** | 20q11.21 | *CHMP4B* | Charged multivesicular body protein 4B | 128866 | 610897 | AD |  |
| **34** | 21q22.3 | *CRYAA* | Alpha A crystallin | 1409 | 123580 | AD/AR |  |
| **35** | 22q11.23 | *CRYBB2* | Beta B2 crystallin | 1415 | 123620 | AD |  |
| **36** | 22q11.23 | *CRYBB3* | Beta B3 crystallin | 1417 | 123630 | AD/AR |  |
| **37** | 22q12.1 | *CRYBB1* | Beta B1 crystallin | 1414 | 6009291 | AD/AR |  |
| **38** | 22q12.1 | *CRYBA4* | Beta A4 crystallin | 1413 | 123631 | AD |  |
| **39** | Xp22.13 | *NHS* | Nance Horan syndrome | 4810 | 300457 | X linked | Nance-Horan syndrome |
