# Supplementary material for: Exome sequencing identifies novel and recurrent mutations in GJA8 and CRYGD associated with inherited cataract
Source: Hum Genomics. 2014 Nov 18;8(1):19. doi: 10.1186/s40246-014-0019-6 (PMC4240822; doi:10.1186/s40246-014-0019-6)
Supplement: Additional file 2: Table S2. — Sample metrics for exome sequencing of trios from families A, B, and C. [file 40246_2014_19_MOESM2_ESM.docx]

**Additional file 2. Table S2.** Sample metrics for exome sequencing of trios from families A, B and C.

| Pedigree/  Exome No. | Disease Status (sex) | Total Mapped Reads  (% total reads) | % Mapped Reads in Exome | Mean Mapped Read Depth for Exome (X) | % Exome ≥10X Coverage | No. Exome SNPs | Non-synon. SNPs | Novel SNPs | Unexpected Gaps |
| --- | --- | --- | --- | --- | --- | --- | --- | --- | --- |
| A-1:1 | Spouse (M) | 81,797,226 (98.04) | 79.13 | 163.9 | 98.25 | 40,239 | 8,608 | 1,526 | none |
| A-1:2 | Affected (F) | 78,499,381 (98.14) | 78.53 | 157.3 | 98.11 | 40,506 | 8,677 | 1,521 | chr1: 5 exons  chrY: 258 exons |
| A-2:1 | Affected (F) | 81,921,824 (98.14) | 78.35 | 164.2 | 98.32 | 40,824 | 8,806 | 1,594 | chr1: 5 exons  chrY: 239 exons |
| B-I:1 | Affected (M) | 78,779,463 (99.23) | 79.07 | 157.9 | 98.22 | 39,604 | 8,480 | 1,512 | chr22: 11 exons |
| B-I:2 | Spouse (F) | 70,962,063 (99.18) | 84.21 | 142.2 | 97.10 | 40,170 | 8,684 | 1,595 | chr14: 5 exons  chrY: 263 exons |
| B-II:1 | Affected (F) | 70,213,795 (99.05) | 77.78 | 140.7 | 97.94 | 40,307 | 8,748 | 1,663 | chr1: 5 exons  chr11: 5 exons  chr22: 8 exons  chrY: 237 exons |
| C-I:1 | Affected (M) | 67,695,709 (99.17) | 77.85 | 135.7 | 80.61 | 34,435 | 7,639 | 1,331 | chr2: 11 exons  chr3: 5 exons  chr4: 6 exons  chr5: 5 exons  chr8: 23 exons  chr10: 5 exons  chr12: 10 exons  chr14: 10 exons  chr19: 5 exons  chr20: 5 exons  chrX: 23 exons |
| C-I:2 | Spouse (F) | 66,919,669 (99.29) | 79.47 | 134.1 | 97.01 | 39,484 | 8,565 | 1,520 | chr1: 5 exons  chr22: 8 exons  chrY: 281 exons |
| C-II:2 | Affected (F) | 73,764,201 (99.30) | 79.22 | 147.8 | 97.17 | 39,359 | 8,563 | 1,511 | chr7: 5 exons  chr8: 10 exons  chrY: 294 exons |
