# Supplementary material for: Exome sequencing identifies novel and recurrent mutations in GJA8 and CRYGD associated with inherited cataract
Source: Hum Genomics. 2014 Nov 18;8(1):19. doi: 10.1186/s40246-014-0019-6 (PMC4240822; doi:10.1186/s40246-014-0019-6)
Supplement: Additional file 3: Table S3. — Exome sequencing variants found in candidate genes for inherited cataract (Additional file 1, Table S1) in family trios A, B, and C. [file 40246_2014_19_MOESM3_ESM.docx]

**Additional file 3. Table S3.** Exome sequencing variants found in candidate genes for inherited cataract (Additional file 1) in family trios A, B, and C.

| **Chr** | **Position (bp)** | **Gene Region** | **Gene Symbol** | **Protein Variant** | **Translation Impact** | **dbSNP ID**  **(rs no.)** | **SIFT value** | **MAF (%)** |  | **Family A I:1** | **Family A I:2** | **Family A II:1** |  | **Family B I:1** | **Family B I:2** | **Family B II:1** |  | **Family C I:1** | **Family C I:2** | **Family C II:2** |
| --- | --- | --- | --- | --- | --- | --- | --- | --- | --- | --- | --- | --- | --- | --- | --- | --- | --- | --- | --- | --- |
| 1 | 16451767 | Exonic | *EPHA2* | p.I958I | synonymous | 3754334 |  | 28.13 |  |  |  |  |  | het | het | hom |  |  |  |  |
| 1 | 16464673 | Exonic | *EPHA2* | p.P329P | synonymous | 2230597 |  | 40.32 |  | het | het | hom |  | hom | het | hom |  | het | het | hom |
| 1 | 16475048 | Exonic | *EPHA2* | p.A216A | synonymous | 34753465 |  | 0.53 |  |  |  |  |  | het |  | het |  |  |  |  |
| 1 | 16475123 | Exonic | *EPHA2* | p.L191L | synonymous | 6678616 |  | 34.57 |  |  |  |  |  | het | het | hom |  | het | het | hom |
| 1 | 16475126 | Exonic | *EPHA2* | p.A190A | synonymous | 6678618 |  | 34.55 |  |  |  |  |  | het | hom | het |  | het | het | hom |
| 1 | 16477467 | Intronic | *EPHA2* |  |  | 138164293 |  | 0.85 |  |  |  |  |  |  |  |  |  | het |  |  |
| 1 | 47882497 | Exonic | *FOXE3* | p.A170A | synonymous | 34082359 |  | 34.46 |  |  | hom | het |  |  | het |  |  |  |  |  |
| **1** | **147380102** | **Exonic** | ***GJA8*** | **p.L7P** | **missense** | **Novel** | **0.00** | **N/A** |  |  |  |  |  | **het** |  | **het** |  |  |  |  |
| **1** | **147380375** | **Exonic** | ***GJA8*** | **p.H98P** | **missense** | **Novel** | **0.00** | **N/A** |  |  |  |  |  |  |  |  |  | **het** |  | **het** |
| 2 | 1643208 | Intronic | *PXDN* |  |  | 7578605 |  | 6.25 |  |  |  |  |  | het |  | het |  |  |  |  |
| 2 | 1647171 | Exonic | *PXDN* | p.V1307V | synonymous | 6730800 |  | 86.30 |  | hom | hom | hom |  |  |  |  |  |  |  |  |
| 2 | 1652660 | Exonic | *PXDN* | p.N964N | synonymous | 3811613 |  | 29.89 |  | het |  |  |  |  |  |  |  | het | het | het |
| 2 | 1652731 | Exonic | *PXDN* | p.V941M | missense | 189824177 | 0.01 | 0.50 |  |  | het | het |  |  | het | het |  |  |  |  |
| 2 | 1652822 | Exonic | *PXDN* | p.I910I | synonymous | 1863135 |  | 90.03 |  | hom | hom | hom |  | het | hom | hom |  | hom | hom | hom |
| 2 | 1664654 | Exonic | *PXDN* | p.N612N | synonymous | 17841813 |  | 70.01 |  | hom | hom | hom |  |  |  |  |  |  |  |  |
| 2 | 1667310 | Intronic | *PXDN* |  |  | 12475392 |  | 82.47 |  | hom | hom | hom |  | het | hom | hom |  | het | hom | hom |
| 2 | 208986637 | Exonic | *CRYGD* | p.R95R | synonymous | 2305430 |  | 91.52 |  | hom | hom | hom |  | het | het |  |  | hom | hom | hom |
| **2** | **208989018** | **Exonic** | ***CRYGD*** | **p.P24T** | **missense** | **28931605** | **0.25** | **N/A** |  |  | **het** | **het** |  |  |  |  |  |  |  |  |
| 2 | 208989037 | Exonic | *CRYGD* | p.Y17Y | synonymous | 2242074 |  | 51.53 |  | het | hom | hom |  |  |  |  |  |  |  |  |
| 2 | 208994274 | Exonic | *CRYGC* | p.R48H | missense | 61751949 | 0.09 | 1.47 |  |  | het |  |  |  |  |  |  |  |  |  |
| 2 | 209007559 | Exonic | *CRYGB* | p.I111L | missense | 796287 | 0.67 | 73.65 |  | hom | hom | hom |  |  |  |  |  | hom | hom | hom |
| 2 | 209010558 | Exonic | *CRYGB* | p.P64P | synonymous | 2854723 |  | 53.71 |  | het | hom | hom |  |  |  |  |  | het | het | het |
| 2 | 209010891 | Promoter | *CRYGB* |  |  | 2289917 |  | 39.86 |  | het | hom | hom |  |  |  |  |  | het | het | het |
| 3 | 45988525 | Intronic | *FYCO1* |  |  | 61751654 |  | 0.76 |  |  |  |  |  |  |  |  |  |  | het | het |
| 3 | 45996761 | Exonic | *FYCO1* | p.L1308L | synonymous | 1463680 |  | 71.93 |  | hom | het | het |  | het | het | hom |  | hom | hom | hom |
| 3 | 46001063 | Intronic | *FYCO1* |  |  | 1532071 |  | 51.40 |  | het |  | het |  |  |  |  |  | hom | hom | hom |
| 3 | 46008087 | Exonic | *FYCO1* | p.C913C | synonymous | 13079869 |  | 11.01 |  | het |  | het |  |  |  |  |  | het |  |  |
| 3 | 46008790 | Exonic | *FYCO1* | p.A679V | missense | 3796375 | 0.12 | 43.10 |  |  |  |  |  | het | het |  |  | het | hom | hom |
| 3 | 46009487 | Exonic | *FYCO1* | p.R447C | missense | 33910087 | 0.00 | 11.02 |  | het |  | het |  |  |  |  |  | het |  |  |
| 3 | 46009491 | Exonic | *FYCO1* | p.L445L | synonymous | 3796376 |  | 19.60 |  | het | het |  |  | het | het | hom |  |  |  |  |
| 3 | 46009864 | Exonic | *FYCO1* | p.G321A | missense | 3733100 | 1.00 | 54.40 |  | het |  | het |  | het | het |  |  | hom | hom | hom |
| 3 | 46010007 | Exonic | *FYCO1* | p.Q273Q | synonymous | 13071283 |  | 11.20 |  | het |  | het |  |  |  |  |  | het |  |  |
| 3 | 46010077 | Exonic | *FYCO1* | p.R250Q | missense | 4683158 | 1.00 | 77.47 |  | het | het | het |  | hom | hom | hom |  | hom | hom | hom |
| 3 | 46014545 | Intronic | *FYCO1* |  |  | 41289622 |  | 11.01 |  | het |  | het |  |  |  |  |  | het |  |  |
| 3 | 46016851 | Intronic | *FYCO1* |  |  | 751552 |  | 43.10 |  |  |  |  |  | het | het |  |  | het | hom | hom |
| 3 | 46021218 | Exonic | *FYCO1* | p.R89R | synonymous | 4682801 |  | 77.15 |  | hom | het | het |  | hom | hom | hom |  | hom | hom | hom |
| 3 | 46026259 | Intronic | *FYCO1* |  |  | 3733097 |  | 41.61 |  |  |  |  |  | het | het |  |  | het | hom | hom |
| 3 | 133167363 | Exonic | *BFSP2* | p.A201A | synonymous | 2276737 |  | 61.42 |  | het | hom | hom |  | het | hom | hom |  | hom | het | hom |
| 3 | 133185834 | Intronic | *BFSP2* |  |  | 2737717 |  | 40.79 |  | het | het | hom |  | het | het | het |  | hom |  | het |
| 3 | 133185849 | Intronic | *BFSP2* |  |  | 10563564 |  | 11.23 |  | het |  |  |  | het |  |  |  |  | het | het |
| 4 | 6292909 | Intronic | *WFS1* |  |  | 9998519 |  | 60.87 |  | het | het |  |  | het | hom | hom |  | het | hom | hom |
| 4 | 6292915 | Intronic | *WFS1* |  |  | 10010131 |  | 60.91 |  | het | het |  |  | het | hom | hom |  | het | hom | hom |
| 4 | 6293550 | Intronic | *WFS1* |  |  | 115561055 |  | 1.34* |  |  |  |  |  |  | het | het |  |  |  |  |
| 4 | 6293696 | Exonic | *WFS1* | p.R228R | synonymous | 1801213 |  | 68.27 |  | het | het |  |  | hom | hom | hom |  | hom | hom | hom |
| 4 | 6302519 | Exonic | *WFS1* | p.V333I | missense | 1801212 | 1.00 | 72.37 |  | hom | het | het |  | hom | hom | hom |  | hom | hom | hom |
| 4 | 6302545 | Exonic | *WFS1* | p.F341F | synonymous | 56072215 |  | 7.35 |  |  |  |  |  | het |  |  |  | het | het |  |
| 4 | 6302707 | Exonic | *WFS1* | p.V395V | synonymous | 1801206 |  | 59.83 |  | het | het |  |  | het | hom | hom |  | het | het | hom |
| 4 | 6302889 | Exonic | *WFS1* | p.R456H | missense | 1801208 | 0.01 | 5.08 |  |  |  |  |  |  |  |  |  |  | het | het |
| 4 | 6303022 | Exonic | *WFS1* | p.N500N | synonymous | 1801214 |  | 60.98 |  | het | het |  |  | het | hom | hom |  | het | het | hom |
| 4 | 6303247 | Exonic | *WFS1* | p.A575A | synonymous | 2230719 |  | 7.24 |  |  |  |  |  | het |  |  |  | het |  |  |
| 4 | 6303354 | Exonic | *WFS1* | p.R611H | missense | 734312 | 0.05 | 54.69 |  |  | het |  |  | het | het | het |  | het |  | het |
| 4 | 6303844 | Exonic | *WFS1* | p.K774K | synonymous | 2230721 |  | 7.04 |  |  |  |  |  | het |  |  |  | het | het |  |
| 4 | 6303955 | Exonic | *WFS1* | p.K811K | synonymous | 1046314 |  | 59.66 |  | het | het |  |  | het | hom | hom |  | het | hom | hom |
| 4 | 6304087 | Exonic | *WFS1* | p.S855S | synonymous | 1046316 |  | 68.70 |  | het | het |  |  | hom | hom | hom |  | hom | hom | hom |
| 5 | 138456815 | Exonic | *SIL1* | p.T51T | synonymous | 3088052 |  | 43.08 |  | het | hom | hom |  | het | het | het |  | het | het | het |
| 5 | 138463538 | 5'UTR | *SIL1* |  |  | 11555154 |  | 7.94 |  |  |  |  |  |  |  |  |  |  | het | het |
| 5 | 138473077 | Intronic | *SIL1* |  |  | 11431215 |  | 100* |  | hom | hom | hom |  | hom | hom | hom |  | hom | hom | hom |
| 6 | 10586823 | Exonic | *GCNT2* | p.R201R | synonymous | 147898876 |  | 0.02 |  | het |  | het |  |  |  |  |  |  |  |  |
| 6 | 10587038 | Exonic | *GCNT2* | p.D272E | missense | 539351 | 1.00 | 99.87* |  | hom | hom | hom |  | hom | hom | hom |  | hom | hom | hom |
| 6 | 10587056 | Exonic | *GCNT2* | p.L278L | synonymous | 71548508 |  | 14.77 |  |  |  |  |  |  | hom | het |  | het |  |  |
| 6 | 10621547 | Intronic | *GCNT2* |  |  | 9460944 |  | 4.47 |  |  |  |  |  | het | hom | het |  |  | het |  |
| 8 | 72111599 | Exonic | *EYA1* | p.H585H | synonymous | 10103397 |  | 30.52 |  |  | hom | het |  | het |  | het |  | het |  | het |
| 8 | 72111678 | Intronic | *EYA1* |  |  | 10090382 |  | 30.41 |  |  | hom | het |  | het |  | het |  | het |  | het |
| 8 | 72111710 | Intronic | *EYA1* |  |  | 10103644 |  | 17.60 |  |  | hom | het |  | het |  | het |  |  |  |  |
| 8 | 72111739 | Intronic | *EYA1* |  |  | 10103852 |  | 45.80 |  |  | hom | het |  | het |  | het |  | het |  | het |
| 8 | 72127764 | Intronic | *EYA1* |  |  | 3735935 |  | 29.20 |  |  | hom | het |  | het |  | het |  | het |  | het |
| 8 | 72129009 | Exonic | *EYA1* | p.G426G | synonymous | Novel |  | N/A |  |  |  |  |  |  |  |  |  | het |  | het |
| 8 | 72211295 | Exonic | *EYA1* | p.T271T | synonymous | 1445398 |  | 8.44 |  |  |  |  |  | het |  | het |  | het |  |  |
| 9 | 100190780 | Exonic | *TDRD7* | p.L11L | synonymous | 1381532 |  | 47.56 |  | het | hom | hom |  |  | het |  |  | het | het | hom |
| 9 | 100194406 | Exonic | *TDRD7* | p.V150A | missense | 2045732 | 0.50 | 47.53 |  | het | hom | hom |  |  | het |  |  | het | het | hom |
| 9 | 100201664 | Intronic | *TDRD7* |  |  | Novel |  | N/A |  |  |  |  |  |  |  |  |  | het | het |  |
| 9 | 100237719 | Exonic | *TDRD7* | p.F712L | missense | 149857246 | 0.29 | 0.03 |  |  |  |  |  |  |  |  |  | het |  |  |
| 10 | 17279377 | 3'UTR | *VIM* |  |  | 112279926 |  | N/A |  |  |  | het |  |  |  |  |  |  |  |  |
| 10 | 91222287 | Exonic | *SLC16A12* | p.W17G | missense | 3740030 | 0.00 | 8.50 |  |  | het | het |  |  |  |  |  |  |  |  |
| 10 | 103991381 | Exonic | *PITX3* | p.I95I | synonymous | 2281983 |  | 61.64 |  | hom | hom | hom |  |  |  |  |  |  |  |  |
| 11 | 31814879 | Intronic | *PAX6* |  |  | 3026384 |  | 18.28* |  |  | het | het |  |  |  |  |  |  |  |  |
| 11 | 31838128 | Intronic | *PAX6* |  |  | 4440995 |  | 24.73 |  | hom | het | het |  |  |  |  |  | het | hom | het |
| 11 | 111781047 | Intronic | *CRYAB* |  |  | 11603779 |  | 30.00 |  |  | het | het |  |  | het | het |  |  |  |  |
| 13 | 20716411 | Exonic | *GJA3* | p.A339A | synonymous | 11617415 |  | 19.86 |  |  | het |  |  |  |  |  |  |  |  |  |
| 13 | 20716533 | Exonic | *GJA3* | p.L299M | missense | 968566 | 0.87 | 99.94 |  | hom | hom | hom |  | hom | hom | hom |  | hom | hom | hom |
| 16 | 67201261 | Intronic | *HSF4* |  |  | 113049652 |  | 0.69* |  |  | het |  |  |  |  |  |  |  |  |  |
| 16 | 79628325 | 3'UTR | *MAF* |  |  | 148849596 |  | 9.18 |  |  |  |  |  |  | hom | het |  | het |  |  |
| 17 | 27579011 | Intronic | *CRYBA1* |  |  | 55885610 |  | 1.19 |  |  |  |  |  | het |  | het |  |  |  |  |
| 17 | 33477242 | Exonic | *UNC45B* | p.K127K | synonymous | 80100968 |  | 9.31 |  |  | het |  |  |  |  |  |  |  |  |  |
| 17 | 33482522 | Intronic | *UNC45B* |  |  | 11869662 |  | 11.99 |  |  |  |  |  | het |  |  |  |  |  | het |
| 17 | 33504194 | Intronic | *UNC45B* |  |  | 78278095 |  | 9 |  |  |  |  |  | het |  |  |  |  | het | het |
| 17 | 33504716 | Intronic | *UNC45B* |  |  | 77782351 |  | 7.73* |  |  |  |  |  | het |  |  |  |  | het | het |
| 17 | 33513591 | 3'UTR | *UNC45B* |  |  | 3744365 |  | 20.21 |  |  | het | het |  |  |  |  |  | het | het | het |
| 17 | 73754248 | Intronic | *GALK1* |  |  | 743554 |  | 14.37 |  | hom |  | het |  | het |  | het |  |  | het | het |
| 17 | 73759039 | Intronic | *GALK1* |  |  | 35008831 |  | 99.64* |  | hom | hom | hom |  | hom | hom | hom |  | hom | hom | hom |
| 17 | 73760626 | Intronic | *GALK1* |  |  | 56038529 |  | 11.02 |  |  | het | het |  |  |  |  |  |  |  |  |
| 17 | 73760820 | Intronic | *GALK1* |  |  | 55715426 |  | 11.47 |  |  | het | het |  |  |  |  |  |  |  |  |
| 19 | 49469087 | Exonic | *FTL* | p.L55L | synonymous | 2230267 |  | 54.29 |  | het |  | het |  |  |  |  |  |  |  |  |
| 19 | 49469233 | Intronic | *FTL* |  |  | Novel |  |  |  | het |  | het |  |  |  |  |  |  |  |  |
| 20 | 17474690 | 3'UTR | *BFSP1* |  |  | 6105762 |  | 52.11 |  | hom | het | hom |  | hom | hom | hom |  | hom | hom | hom |
| 20 | 17474710 | 3'UTR | *BFSP1* |  |  | 41276390 |  | 0.40 |  |  |  |  |  | het |  | het |  |  |  |  |
| 20 | 17474791 | Exonic | *BFSP1* | p.T642T | synonymous | 6080717 |  | 28.88 |  |  | het | het |  | het | het | het |  | het | hom | hom |
| 20 | 17474968 | Exonic | *BFSP1* | p.P583P | synonymous | 6080718 |  | 70.99 |  | hom | hom | hom |  | hom | hom | hom |  | hom | hom | hom |
| 20 | 17475217 | Exonic | *BFSP1* | p.A500A | synonymous | 6136118 |  | 23.58 |  | hom |  | het |  | hom | het | hom |  | het |  |  |
| 20 | 17477592 | Exonic | *BFSP1* | p.G345S | missense | 6080719 | 0.83 | 21.17 |  | hom |  | het |  | het | het | het |  | het |  |  |
| 20 | 17477751 | Intronic | *BFSP1* |  |  | 6044850 |  | 55.17* |  | hom | het | het |  | hom | het | hom |  | het |  |  |
| 20 | 17479617 | Exonic | *BFSP1* | p.N268N | synonymous | 11537702 |  | 7.87 |  |  |  |  |  |  |  |  |  |  | het | het |
| 20 | 17489733 | Intronic | *BFSP1* |  |  | 2281207 |  | 25.74 |  |  | het |  |  |  |  |  |  | het |  | het |
| 20 | 17492755 | Intronic | *BFSP1* |  |  | 1559956 |  | 24.29 |  | het | het |  |  | het |  | het |  | het | het | het |
| 21 | 44589215 | Exonic | *CRYAA* | p.D2D | synonymous | 872331 |  | 60.06 |  | hom | het | het |  |  | het |  |  | het |  |  |
| 22 | 25597331 | Intronic | *CRYBB3* |  |  | 2269672 |  | 52.44 |  |  | hom | het |  |  | hom | het |  | het | het | het |
| 22 | 25601196 | Exonic | *CRYBB3* | p.H113D | missense | 9608378 | 1.00 | 63.08 |  | het | hom | het |  |  | hom | het |  | hom | het | het |
| 22 | 27009128 | Intronic | *CRYBB1* |  |  | 4822751 |  | 18.44* |  |  | het |  |  | het |  |  |  | het |  | het |
| 22 | 27021425 | Intronic | *CRYBA4* |  |  | 4276 |  | 47.97 |  | het |  | het |  |  | het | het |  |  |  |  |
| 22 | 27021457 | Exonic | *CRYBA4* | p.F57F | synonymous | 5761637 |  | 84.71 |  | hom | het | het |  | hom | hom | hom |  | hom |  | het |
| X | 17705850 | Intronic | *NHS* |  |  | 5901624 |  | 100.00 |  | hom | hom | hom |  | hom | hom | hom |  | hom | hom | hom |
| X | 17746244 | Exonic | *NHS* | p.F1319L | missense | 3747295 | 1.00 | 2.68 |  |  |  |  |  | hom |  | het |  |  |  |  |
