# Supplementary material for: Exome sequencing identifies novel and recurrent mutations in GJA8 and CRYGD associated with inherited cataract
Source: Hum Genomics. 2014 Nov 18;8(1):19. doi: 10.1186/s40246-014-0019-6 (PMC4240822; doi:10.1186/s40246-014-0019-6)
Supplement: Additional file 4: Table S4. — Recurrence of the p.Pro24Thr mutation in CRYGD associated with autosomal dominant cataract. [file 40246_2014_19_MOESM4_ESM.docx]

**Additional file 4. Table S4.** Recurrence of p.Pro24Thr mutation in *CRYGD* associated with autosomal dominant cataract.

| **Country of origin** | **Cataract phenotype** | **No. of affected**  **family members** | **Reference** |
| --- | --- | --- | --- |
| India | Lamellar | 2 | [Santhiya ST](http://www.ncbi.nlm.nih.gov/pubmed?term=Santhiya%20ST%5BAuthor%5D&cauthor=true&cauthor_uid=12011157), [Shyam Manohar M](http://www.ncbi.nlm.nih.gov/pubmed?term=Shyam%20Manohar%20M%5BAuthor%5D&cauthor=true&cauthor_uid=12011157), [Rawlley D](http://www.ncbi.nlm.nih.gov/pubmed?term=Rawlley%20D%5BAuthor%5D&cauthor=true&cauthor_uid=12011157), [Vijayalakshmi P](http://www.ncbi.nlm.nih.gov/pubmed?term=Vijayalakshmi%20P%5BAuthor%5D&cauthor=true&cauthor_uid=12011157), Namperumalsamy P, [Gopinath PM](http://www.ncbi.nlm.nih.gov/pubmed?term=Gopinath%20PM%5BAuthor%5D&cauthor=true&cauthor_uid=12011157), [Löster J](http://www.ncbi.nlm.nih.gov/pubmed?term=L%C3%B6ster%20J%5BAuthor%5D&cauthor=true&cauthor_uid=12011157), [Graw J](http://www.ncbi.nlm.nih.gov/pubmed?term=Graw%20J%5BAuthor%5D&cauthor=true&cauthor_uid=12011157): Novel mutations in the gamma-crystallin genes cause autosomal dominant congenital cataracts: *J Med Genet* 2002, 39:352-358. |
| Morocco | Cerulean | 19 | [Nandrot E](http://www.ncbi.nlm.nih.gov/pubmed?term=Nandrot%20E%5BAuthor%5D&cauthor=true&cauthor_uid=12676897), [Slingsby C](http://www.ncbi.nlm.nih.gov/pubmed?term=Slingsby%20C%5BAuthor%5D&cauthor=true&cauthor_uid=12676897), [Basak A](http://www.ncbi.nlm.nih.gov/pubmed?term=Basak%20A%5BAuthor%5D&cauthor=true&cauthor_uid=12676897), [Cherif-Chefchaouni M](http://www.ncbi.nlm.nih.gov/pubmed?term=Cherif-Chefchaouni%20M%5BAuthor%5D&cauthor=true&cauthor_uid=12676897), [Benazzouz B](http://www.ncbi.nlm.nih.gov/pubmed?term=Benazzouz%20B%5BAuthor%5D&cauthor=true&cauthor_uid=12676897), [Hajaji Y](http://www.ncbi.nlm.nih.gov/pubmed?term=Hajaji%20Y%5BAuthor%5D&cauthor=true&cauthor_uid=12676897), [Boutayeb S](http://www.ncbi.nlm.nih.gov/pubmed?term=Boutayeb%20S%5BAuthor%5D&cauthor=true&cauthor_uid=12676897), [Gribouval O](http://www.ncbi.nlm.nih.gov/pubmed?term=Gribouval%20O%5BAuthor%5D&cauthor=true&cauthor_uid=12676897), [Arbogast L](http://www.ncbi.nlm.nih.gov/pubmed?term=Arbogast%20L%5BAuthor%5D&cauthor=true&cauthor_uid=12676897), [Berraho A](http://www.ncbi.nlm.nih.gov/pubmed?term=Berraho%20A%5BAuthor%5D&cauthor=true&cauthor_uid=12676897), [Abitbol M](http://www.ncbi.nlm.nih.gov/pubmed?term=Abitbol%20M%5BAuthor%5D&cauthor=true&cauthor_uid=12676897), [Hilal L](http://www.ncbi.nlm.nih.gov/pubmed?term=Hilal%20L%5BAuthor%5D&cauthor=true&cauthor_uid=12676897): Gamma-D crystallin gene (CRYGD) mutation causes autosomal dominant congenital cerulean cataracts. *J Med Genet* 2003, 40:262-267. |
| Australia | Flaky, silica-like | 20 | [Burdon KP](http://www.ncbi.nlm.nih.gov/pubmed?term=Burdon%20KP%5BAuthor%5D&cauthor=true&cauthor_uid=14693780), [Wirth MG](http://www.ncbi.nlm.nih.gov/pubmed?term=Wirth%20MG%5BAuthor%5D&cauthor=true&cauthor_uid=14693780), [Mackey DA](http://www.ncbi.nlm.nih.gov/pubmed?term=Mackey%20DA%5BAuthor%5D&cauthor=true&cauthor_uid=14693780), [Russell-Eggitt IM](http://www.ncbi.nlm.nih.gov/pubmed?term=Russell-Eggitt%20IM%5BAuthor%5D&cauthor=true&cauthor_uid=14693780), [Craig JE](http://www.ncbi.nlm.nih.gov/pubmed?term=Craig%20JE%5BAuthor%5D&cauthor=true&cauthor_uid=14693780), [Elder JE](http://www.ncbi.nlm.nih.gov/pubmed?term=Elder%20JE%5BAuthor%5D&cauthor=true&cauthor_uid=14693780), [Dickinson JL](http://www.ncbi.nlm.nih.gov/pubmed?term=Dickinson%20JL%5BAuthor%5D&cauthor=true&cauthor_uid=14693780), [Sale MM](http://www.ncbi.nlm.nih.gov/pubmed?term=Sale%20MM%5BAuthor%5D&cauthor=true&cauthor_uid=14693780): Investigation of crystallin genes in familial cataract, and report of two disease associated mutations. *Br J Ophthalmol* 2004, 88:79-83. |
| Europe | Coral-like | 7 | [Mackay DS](http://www.ncbi.nlm.nih.gov/pubmed?term=Mackay%20DS%5BAuthor%5D&cauthor=true&cauthor_uid=15041957), [Andley UP](http://www.ncbi.nlm.nih.gov/pubmed?term=Andley%20UP%5BAuthor%5D&cauthor=true&cauthor_uid=15041957), [Shiels A](http://www.ncbi.nlm.nih.gov/pubmed?term=Shiels%20A%5BAuthor%5D&cauthor=true&cauthor_uid=15041957): A missense mutation in the gammaD crystallin gene (CRYGD) associated with autosomal dominant "coral-like" cataract linked to chromosome 2q. *Mol Vis* 2004, 10:155-162. |
| China | Fasciculiform | 13 | [Shentu X](http://www.ncbi.nlm.nih.gov/pubmed?term=Shentu%20X%5BAuthor%5D&cauthor=true&cauthor_uid=15064679), [Yao K](http://www.ncbi.nlm.nih.gov/pubmed?term=Yao%20K%5BAuthor%5D&cauthor=true&cauthor_uid=15064679), [Xu W](http://www.ncbi.nlm.nih.gov/pubmed?term=Xu%20W%5BAuthor%5D&cauthor=true&cauthor_uid=15064679), [Zheng S](http://www.ncbi.nlm.nih.gov/pubmed?term=Zheng%20S%5BAuthor%5D&cauthor=true&cauthor_uid=15064679), [Hu S](http://www.ncbi.nlm.nih.gov/pubmed?term=Hu%20S%5BAuthor%5D&cauthor=true&cauthor_uid=15064679), [Gong X](http://www.ncbi.nlm.nih.gov/pubmed?term=Gong%20X%5BAuthor%5D&cauthor=true&cauthor_uid=15064679): Special fasciculiform cataract caused by a mutation in the gammaD-crystallin gene. *Mol Vis* 2004, 10:233-239. |
| China | Coralliform | 11 | [Xu WZ](http://www.ncbi.nlm.nih.gov/pubmed?term=Xu%20WZ%5BAuthor%5D&cauthor=true&cauthor_uid=15161542), [Zheng S](http://www.ncbi.nlm.nih.gov/pubmed?term=Zheng%20S%5BAuthor%5D&cauthor=true&cauthor_uid=15161542), [Xu SJ](http://www.ncbi.nlm.nih.gov/pubmed?term=Xu%20SJ%5BAuthor%5D&cauthor=true&cauthor_uid=15161542), [Huang W](http://www.ncbi.nlm.nih.gov/pubmed?term=Huang%20W%5BAuthor%5D&cauthor=true&cauthor_uid=15161542), [Yao K](http://www.ncbi.nlm.nih.gov/pubmed?term=Yao%20K%5BAuthor%5D&cauthor=true&cauthor_uid=15161542), [Zhang SZ](http://www.ncbi.nlm.nih.gov/pubmed?term=Zhang%20SZ%5BAuthor%5D&cauthor=true&cauthor_uid=15161542): Autosomal dominant coralliform cataract related to a missense mutation of the gammaD-crystallin gene. *Chin Med J (Engl)* 2004, 117:727-732. |
| Mid-Asia | Polymorphic | 6 | [Plotnikova OV](http://www.ncbi.nlm.nih.gov/pubmed?term=Plotnikova%20OV%5BAuthor%5D&cauthor=true&cauthor_uid=17564961), [Kondrashov FA](http://www.ncbi.nlm.nih.gov/pubmed?term=Kondrashov%20FA%5BAuthor%5D&cauthor=true&cauthor_uid=17564961), [Vlasov PK](http://www.ncbi.nlm.nih.gov/pubmed?term=Vlasov%20PK%5BAuthor%5D&cauthor=true&cauthor_uid=17564961), [Grigorenko AP](http://www.ncbi.nlm.nih.gov/pubmed?term=Grigorenko%20AP%5BAuthor%5D&cauthor=true&cauthor_uid=17564961), [Ginter EK](http://www.ncbi.nlm.nih.gov/pubmed?term=Ginter%20EK%5BAuthor%5D&cauthor=true&cauthor_uid=17564961), [Rogaev EI](http://www.ncbi.nlm.nih.gov/pubmed?term=Rogaev%20EI%5BAuthor%5D&cauthor=true&cauthor_uid=17564961): Conversion and compensatory evolution of the gamma-crystallin genes and identification of a cataractogenic mutation that reverses the sequence of the human CRYGD gene to an ancestral state. *Am J Hum Genet* 2007, 81:32-43. |
| Saudi Arabia | Cerulean/Coralliform 1  Cerulean/Coralliform 2 | 5  1 | [Khan AO](http://www.ncbi.nlm.nih.gov/pubmed?term=Khan%20AO%5BAuthor%5D&cauthor=true&cauthor_uid=19633732), [Aldahmesh MA](http://www.ncbi.nlm.nih.gov/pubmed?term=Aldahmesh%20MA%5BAuthor%5D&cauthor=true&cauthor_uid=19633732), [Ghadhfan FE](http://www.ncbi.nlm.nih.gov/pubmed?term=Ghadhfan%20FE%5BAuthor%5D&cauthor=true&cauthor_uid=19633732), Al-Mesfer S, [Alkuraya FS](http://www.ncbi.nlm.nih.gov/pubmed?term=Alkuraya%20FS%5BAuthor%5D&cauthor=true&cauthor_uid=19633732): Founder heterozygous P23T CRYGD mutation associated with cerulean (and coralliform) cataract in 2 Saudi families. *Mol Vis* 2009, 15:1407-1411. |
| China | Coralliform | 6 | [Zhang LY](http://www.ncbi.nlm.nih.gov/pubmed?term=Zhang%20LY%5BAuthor%5D&cauthor=true&cauthor_uid=19668596), [Gong B](http://www.ncbi.nlm.nih.gov/pubmed?term=Gong%20B%5BAuthor%5D&cauthor=true&cauthor_uid=19668596), [Tong JP](http://www.ncbi.nlm.nih.gov/pubmed?term=Tong%20JP%5BAuthor%5D&cauthor=true&cauthor_uid=19668596), [Fan DS](http://www.ncbi.nlm.nih.gov/pubmed?term=Fan%20DS%5BAuthor%5D&cauthor=true&cauthor_uid=19668596), [Chiang SW](http://www.ncbi.nlm.nih.gov/pubmed?term=Chiang%20SW%5BAuthor%5D&cauthor=true&cauthor_uid=19668596), [Lou D](http://www.ncbi.nlm.nih.gov/pubmed?term=Lou%20D%5BAuthor%5D&cauthor=true&cauthor_uid=19668596), [Lam DS](http://www.ncbi.nlm.nih.gov/pubmed?term=Lam%20DS%5BAuthor%5D&cauthor=true&cauthor_uid=19668596), [Yam GH](http://www.ncbi.nlm.nih.gov/pubmed?term=Yam%20GH%5BAuthor%5D&cauthor=true&cauthor_uid=19668596), [Pang CP](http://www.ncbi.nlm.nih.gov/pubmed?term=Pang%20CP%5BAuthor%5D&cauthor=true&cauthor_uid=19668596): A novel gammaD-crystallin mutation causes mild changes in protein properties but leads to congenital coralliform cataract. *Mol Vis* 2009, 15:1521-1529. |
| China | Coralliform 1  Coralliform 2 | 3  7 | [Yang G](http://www.ncbi.nlm.nih.gov/pubmed?term=Yang%20G%5BAuthor%5D&cauthor=true&cauthor_uid=21552497), [Xiong C](http://www.ncbi.nlm.nih.gov/pubmed?term=Xiong%20C%5BAuthor%5D&cauthor=true&cauthor_uid=21552497), [Li S](http://www.ncbi.nlm.nih.gov/pubmed?term=Li%20S%5BAuthor%5D&cauthor=true&cauthor_uid=21552497), [Wang Y](http://www.ncbi.nlm.nih.gov/pubmed?term=Wang%20Y%5BAuthor%5D&cauthor=true&cauthor_uid=21552497), Zhao J: A recurrent mutation in CRYGD is associated with autosomal dominant congenital coralliform cataract in two unrelated Chinese families. *Mol Vis* 2011, 17:1085-1089. |
| India | Acueliform | 20 | [Vanita V](http://www.ncbi.nlm.nih.gov/pubmed?term=Vanita%20V%5BAuthor%5D&cauthor=true&cauthor_uid=22669729), [Singh D](http://www.ncbi.nlm.nih.gov/pubmed?term=Singh%20D%5BAuthor%5D&cauthor=true&cauthor_uid=22669729): A missense mutation in CRYGD linked with autosomal dominant congenital cataract of aculeiform type: *Mol Cell Biochem* 2012, 368:167-172. |
| China | Coralliform | 10 | [Jia X](http://www.ncbi.nlm.nih.gov/pubmed?term=Jia%20X%5BAuthor%5D&cauthor=true&cauthor_uid=24103489), [Zhang F](http://www.ncbi.nlm.nih.gov/pubmed?term=Zhang%20F%5BAuthor%5D&cauthor=true&cauthor_uid=24103489), [Bai J](http://www.ncbi.nlm.nih.gov/pubmed?term=Bai%20J%5BAuthor%5D&cauthor=true&cauthor_uid=24103489), [Gao L](http://www.ncbi.nlm.nih.gov/pubmed?term=Gao%20L%5BAuthor%5D&cauthor=true&cauthor_uid=24103489), [Zhang X](http://www.ncbi.nlm.nih.gov/pubmed?term=Zhang%20X%5BAuthor%5D&cauthor=true&cauthor_uid=24103489), [Sun H](http://www.ncbi.nlm.nih.gov/pubmed?term=Sun%20H%5BAuthor%5D&cauthor=true&cauthor_uid=24103489), [Sun D](http://www.ncbi.nlm.nih.gov/pubmed?term=Sun%20D%5BAuthor%5D&cauthor=true&cauthor_uid=24103489), Guan R, [Sun W](http://www.ncbi.nlm.nih.gov/pubmed?term=Sun%20W%5BAuthor%5D&cauthor=true&cauthor_uid=24103489), [Xu L](http://www.ncbi.nlm.nih.gov/pubmed?term=Xu%20L%5BAuthor%5D&cauthor=true&cauthor_uid=24103489), [Yue Z](http://www.ncbi.nlm.nih.gov/pubmed?term=Yue%20Z%5BAuthor%5D&cauthor=true&cauthor_uid=24103489), [Yu Y](http://www.ncbi.nlm.nih.gov/pubmed?term=Yu%20Y%5BAuthor%5D&cauthor=true&cauthor_uid=24103489), [Fu S](http://www.ncbi.nlm.nih.gov/pubmed?term=Fu%20S%5BAuthor%5D&cauthor=true&cauthor_uid=24103489): Combinational analysis of linkage and exome sequencing identifies the causative mutation in a Chinese family with congenital cataract. *BMC Med Genet* 2013, 14:107. |
| USA | Coralliform | 3 | This study |
