# Supplementary material for: Exome sequencing identifies novel and recurrent mutations in GJA8 and CRYGD associated with inherited cataract
Source: Hum Genomics. 2014 Nov 18;8(1):19. doi: 10.1186/s40246-014-0019-6 (PMC4240822; doi:10.1186/s40246-014-0019-6)
Supplement: Additional file 5: Table S5. — Mutation profile of GJA8. [file 40246_2014_19_MOESM5_ESM.docx]

**Additional file 5. Table S5.** Mutation profile of *GJA8*.

|  | **cDNA change** | **Protein**  **change** | **SIFT score** | **PolyPhen-2 score** | **Protein domain** | **Inherit-ance** | **Country of Origin** | **Cataract phenotype**  **(associated phenotype)** | **Reference** |
| --- | --- | --- | --- | --- | --- | --- | --- | --- | --- |
| 1 | c.20C>T | p.Leu7Pro | 0.00 | 0.991 | N-Term | AD | USA |  | This study |
| 2 | c.68G>C | p.Arg23Thr | 0.00 | 1.00 | N-Term | AD | Iran | Nuclear | Willoughby CE, Arab S, Gandhi R, [Zeinali S](http://www.ncbi.nlm.nih.gov/pubmed?term=Zeinali%20S%5BAuthor%5D&cauthor=true&cauthor_uid=14627691), [Arab S](http://www.ncbi.nlm.nih.gov/pubmed?term=Arab%20S%5BAuthor%5D&cauthor=true&cauthor_uid=14627691), [Luk D](http://www.ncbi.nlm.nih.gov/pubmed?term=Luk%20D%5BAuthor%5D&cauthor=true&cauthor_uid=14627691), Billingsley G, [Munier FL](http://www.ncbi.nlm.nih.gov/pubmed?term=Munier%20FL%5BAuthor%5D&cauthor=true&cauthor_uid=14627691), [Héon E](http://www.ncbi.nlm.nih.gov/pubmed?term=H%C3%A9on%20E%5BAuthor%5D&cauthor=true&cauthor_uid=14627691): A novel GJA8 mutation in an Iranian family with progressive autosomal dominant congenital nuclear cataract. *J Med Genet* 2003, 40:e124. |
| 3 | c.92T>C | p.Ile31Thr | 0.00 | 0.999 | TM-1 | AD | China | Congenital nuclear | [Wang K](http://www.ncbi.nlm.nih.gov/pubmed?term=Wang%20K%5BAuthor%5D&cauthor=true&cauthor_uid=20019893), [Wang B](http://www.ncbi.nlm.nih.gov/pubmed?term=Wang%20B%5BAuthor%5D&cauthor=true&cauthor_uid=20019893), [Wang J](http://www.ncbi.nlm.nih.gov/pubmed?term=Wang%20J%5BAuthor%5D&cauthor=true&cauthor_uid=20019893), [Zhou S](http://www.ncbi.nlm.nih.gov/pubmed?term=Zhou%20S%5BAuthor%5D&cauthor=true&cauthor_uid=20019893), [Yun B](http://www.ncbi.nlm.nih.gov/pubmed?term=Yun%20B%5BAuthor%5D&cauthor=true&cauthor_uid=20019893), [Suo P](http://www.ncbi.nlm.nih.gov/pubmed?term=Suo%20P%5BAuthor%5D&cauthor=true&cauthor_uid=20019893), [Cheng J](http://www.ncbi.nlm.nih.gov/pubmed?term=Cheng%20J%5BAuthor%5D&cauthor=true&cauthor_uid=20019893), Ma X, [Zhu S](http://www.ncbi.nlm.nih.gov/pubmed?term=Zhu%20S%5BAuthor%5D&cauthor=true&cauthor_uid=20019893): A novel GJA8 mutation (p.I31T) causing autosomal dominant congenital cataract in a Chinese family. *Mol Vis* 2009, 15:2813-2820. |
| 4 | c.116C>G | p.Thr39Arg | 0.00 | 1.000 | TM-1 | Sporadic | China | Congenital (microcornea) | [Sun W](http://www.ncbi.nlm.nih.gov/pubmed?term=Sun%20W%5BAuthor%5D&cauthor=true&cauthor_uid=21686328), [Xiao X](http://www.ncbi.nlm.nih.gov/pubmed?term=Xiao%20X%5BAuthor%5D&cauthor=true&cauthor_uid=21686328), [Li S](http://www.ncbi.nlm.nih.gov/pubmed?term=Li%20S%5BAuthor%5D&cauthor=true&cauthor_uid=21686328), [Guo X](http://www.ncbi.nlm.nih.gov/pubmed?term=Guo%20X%5BAuthor%5D&cauthor=true&cauthor_uid=21686328), [Zhang Q](http://www.ncbi.nlm.nih.gov/pubmed?term=Zhang%20Q%5BAuthor%5D&cauthor=true&cauthor_uid=21686328): Mutational screening of six genes in Chinese patients with congenital cataract and microcornea. *Mol Vis* 2011, 17:1508-1513. |
| 5 | c.131T>A | p.Val44Glu | 0.00 | 1.000 | TM-1 | AD | India | Total  (microcornea, mild myopia) | [Devi RR](http://www.ncbi.nlm.nih.gov/pubmed?term=Devi%20RR%5BAuthor%5D&cauthor=true&cauthor_uid=16604058), [Vijayalakshmi P](http://www.ncbi.nlm.nih.gov/pubmed?term=Vijayalakshmi%20P%5BAuthor%5D&cauthor=true&cauthor_uid=16604058): Novel mutations in GJA8 associated with autosomal dominant congenital cataract and microcornea. *Mol Vis* 2006, 12:190-195. |
| 6 | c.134G>C | p.Trp45Ser | 0.00 | 1.000 | TM-1 | AD | India | "Jellyfish-like" (microcornea) | [Vanita V](http://www.ncbi.nlm.nih.gov/pubmed?term=Vanita%20V%5BAuthor%5D&cauthor=true&cauthor_uid=18334946), [Singh JR](http://www.ncbi.nlm.nih.gov/pubmed?term=Singh%20JR%5BAuthor%5D&cauthor=true&cauthor_uid=18334946), [Singh D](http://www.ncbi.nlm.nih.gov/pubmed?term=Singh%20D%5BAuthor%5D&cauthor=true&cauthor_uid=18334946), [Varon R](http://www.ncbi.nlm.nih.gov/pubmed?term=Varon%20R%5BAuthor%5D&cauthor=true&cauthor_uid=18334946), [Sperling K](http://www.ncbi.nlm.nih.gov/pubmed?term=Sperling%20K%5BAuthor%5D&cauthor=true&cauthor_uid=18334946): A novel mutation in GJA8 associated with jellyfish-like cataract in a family of Indian origin. *Mol Vis* 2008, 14:323-326. |
| 7 | c.137G>T | p.Gly46Val | 0.00 | 1.000 | TM-1 |  | Palestine | Total | [Minogue PJ](http://www.ncbi.nlm.nih.gov/pubmed?term=Minogue%20PJ%5BAuthor%5D&cauthor=true&cauthor_uid=19684000), [Tong JJ](http://www.ncbi.nlm.nih.gov/pubmed?term=Tong%20JJ%5BAuthor%5D&cauthor=true&cauthor_uid=19684000), [Arora A](http://www.ncbi.nlm.nih.gov/pubmed?term=Arora%20A%5BAuthor%5D&cauthor=true&cauthor_uid=19684000), [Russell-Eggitt I](http://www.ncbi.nlm.nih.gov/pubmed?term=Russell-Eggitt%20I%5BAuthor%5D&cauthor=true&cauthor_uid=19684000), [Hunt DM](http://www.ncbi.nlm.nih.gov/pubmed?term=Hunt%20DM%5BAuthor%5D&cauthor=true&cauthor_uid=19684000), [Moore AT](http://www.ncbi.nlm.nih.gov/pubmed?term=Moore%20AT%5BAuthor%5D&cauthor=true&cauthor_uid=19684000), [Ebihara L](http://www.ncbi.nlm.nih.gov/pubmed?term=Ebihara%20L%5BAuthor%5D&cauthor=true&cauthor_uid=19684000), [Beyer EC](http://www.ncbi.nlm.nih.gov/pubmed?term=Beyer%20EC%5BAuthor%5D&cauthor=true&cauthor_uid=19684000), [Berthoud VM](http://www.ncbi.nlm.nih.gov/pubmed?term=Berthoud%20VM%5BAuthor%5D&cauthor=true&cauthor_uid=19684000): A mutant connexin50 with enhanced hemichannel function leads to cell death. *Invest Ophthalmol Vis Sci* 2009, 50:5837-5845. |
| 8 | c.136G>A | p.Gly46Arg | 0.00 | 1.000 | TM-1 | AD | China | Congenital (microcornea) | [Sun W](http://www.ncbi.nlm.nih.gov/pubmed?term=Sun%20W%5BAuthor%5D&cauthor=true&cauthor_uid=21686328), [Xiao X](http://www.ncbi.nlm.nih.gov/pubmed?term=Xiao%20X%5BAuthor%5D&cauthor=true&cauthor_uid=21686328), [Li S](http://www.ncbi.nlm.nih.gov/pubmed?term=Li%20S%5BAuthor%5D&cauthor=true&cauthor_uid=21686328), [Guo X](http://www.ncbi.nlm.nih.gov/pubmed?term=Guo%20X%5BAuthor%5D&cauthor=true&cauthor_uid=21686328), [Zhang Q](http://www.ncbi.nlm.nih.gov/pubmed?term=Zhang%20Q%5BAuthor%5D&cauthor=true&cauthor_uid=21686328): Mutational screening of six genes in Chinese patients with congenital cataract and microcornea. *Mol Vis* 2011, 17:1508-1513. |
| 9 | c.139G>T | p.Asp47Tyr | 0.00 | 1.000 | EC-1 | AD | China |  | [Lin Y](http://www.ncbi.nlm.nih.gov/pubmed?term=Lin%20Y%5BAuthor%5D&cauthor=true&cauthor_uid=18247306), [Liu NN](http://www.ncbi.nlm.nih.gov/pubmed?term=Liu%20NN%5BAuthor%5D&cauthor=true&cauthor_uid=18247306), [Lei CT](http://www.ncbi.nlm.nih.gov/pubmed?term=Lei%20CT%5BAuthor%5D&cauthor=true&cauthor_uid=18247306), [Fan YC](http://www.ncbi.nlm.nih.gov/pubmed?term=Fan%20YC%5BAuthor%5D&cauthor=true&cauthor_uid=18247306), [Liu XQ](http://www.ncbi.nlm.nih.gov/pubmed?term=Liu%20XQ%5BAuthor%5D&cauthor=true&cauthor_uid=18247306), [Yang Y](http://www.ncbi.nlm.nih.gov/pubmed?term=Yang%20Y%5BAuthor%5D&cauthor=true&cauthor_uid=18247306), [Wang JF](http://www.ncbi.nlm.nih.gov/pubmed?term=Wang%20JF%5BAuthor%5D&cauthor=true&cauthor_uid=18247306), [Liu B](http://www.ncbi.nlm.nih.gov/pubmed?term=Liu%20B%5BAuthor%5D&cauthor=true&cauthor_uid=18247306), [Yang ZL](http://www.ncbi.nlm.nih.gov/pubmed?term=Yang%20ZL%5BAuthor%5D&cauthor=true&cauthor_uid=18247306): [A novel GJA8 mutation in a Chinese family with autosomal dominant congenital cataract]. *Zhonghua Yi Xue Yi Chuan Xue Za Zhi* 2008, 25:59-62. |
| 10 | c.139G>C | p.Asp47His | 0.00 | 1.000 | EC-1 | AD | China |  | [Li J](http://www.ncbi.nlm.nih.gov/pubmed?term=Li%20J%5BAuthor%5D&cauthor=true&cauthor_uid=23592913), [Wang Q](http://www.ncbi.nlm.nih.gov/pubmed?term=Wang%20Q%5BAuthor%5D&cauthor=true&cauthor_uid=23592913), [Fu Q](http://www.ncbi.nlm.nih.gov/pubmed?term=Fu%20Q%5BAuthor%5D&cauthor=true&cauthor_uid=23592913), [Zhu Y](http://www.ncbi.nlm.nih.gov/pubmed?term=Zhu%20Y%5BAuthor%5D&cauthor=true&cauthor_uid=23592913), [Zhai Y](http://www.ncbi.nlm.nih.gov/pubmed?term=Zhai%20Y%5BAuthor%5D&cauthor=true&cauthor_uid=23592913), [Yu Y](http://www.ncbi.nlm.nih.gov/pubmed?term=Yu%20Y%5BAuthor%5D&cauthor=true&cauthor_uid=23592913), [Zhang K](http://www.ncbi.nlm.nih.gov/pubmed?term=Zhang%20K%5BAuthor%5D&cauthor=true&cauthor_uid=23592913), [Yao K](http://www.ncbi.nlm.nih.gov/pubmed?term=Yao%20K%5BAuthor%5D&cauthor=true&cauthor_uid=23592913): A novel connexin 50 gene (gap junction protein, alpha 8) mutation associated with congenital nuclear and zonular pulverulent cataract. *Mol Vis* 2013, 19:767-774. |
| 11 | c.139G>A | p.Asp47Asn | 0.00 | 1.000 | EC-1 | AD | UK | Nuclear pulverulent | [Arora A](http://www.ncbi.nlm.nih.gov/pubmed?term=Arora%20A%5BAuthor%5D&cauthor=true&cauthor_uid=18006672), [Minogue PJ](http://www.ncbi.nlm.nih.gov/pubmed?term=Minogue%20PJ%5BAuthor%5D&cauthor=true&cauthor_uid=18006672), [Liu X](http://www.ncbi.nlm.nih.gov/pubmed?term=Liu%20X%5BAuthor%5D&cauthor=true&cauthor_uid=18006672), [Addison PK](http://www.ncbi.nlm.nih.gov/pubmed?term=Addison%20PK%5BAuthor%5D&cauthor=true&cauthor_uid=18006672), [Russel-Eggitt I](http://www.ncbi.nlm.nih.gov/pubmed?term=Russel-Eggitt%20I%5BAuthor%5D&cauthor=true&cauthor_uid=18006672), [Webster AR](http://www.ncbi.nlm.nih.gov/pubmed?term=Webster%20AR%5BAuthor%5D&cauthor=true&cauthor_uid=18006672), [Hunt DM](http://www.ncbi.nlm.nih.gov/pubmed?term=Hunt%20DM%5BAuthor%5D&cauthor=true&cauthor_uid=18006672), [Ebihara L](http://www.ncbi.nlm.nih.gov/pubmed?term=Ebihara%20L%5BAuthor%5D&cauthor=true&cauthor_uid=18006672), [Beyer EC](http://www.ncbi.nlm.nih.gov/pubmed?term=Beyer%20EC%5BAuthor%5D&cauthor=true&cauthor_uid=18006672), [Berthoud VM](http://www.ncbi.nlm.nih.gov/pubmed?term=Berthoud%20VM%5BAuthor%5D&cauthor=true&cauthor_uid=18006672), [Moore AT](http://www.ncbi.nlm.nih.gov/pubmed?term=Moore%20AT%5BAuthor%5D&cauthor=true&cauthor_uid=18006672): A novel connexin50 mutation associated with congenital nuclear pulverulent cataracts. *J Med Genet* 2008, 45:155-160. |
| 12 |  |  |  |  |  | AD(IP) | China | Nuclear | [He W](http://www.ncbi.nlm.nih.gov/pubmed?term=He%20W%5BAuthor%5D&cauthor=true&cauthor_uid=21174522), [Li X](http://www.ncbi.nlm.nih.gov/pubmed?term=Li%20X%5BAuthor%5D&cauthor=true&cauthor_uid=21174522), [Chen J](http://www.ncbi.nlm.nih.gov/pubmed?term=Chen%20J%5BAuthor%5D&cauthor=true&cauthor_uid=21174522), [Xu L](http://www.ncbi.nlm.nih.gov/pubmed?term=Xu%20L%5BAuthor%5D&cauthor=true&cauthor_uid=21174522), [Zhang F](http://www.ncbi.nlm.nih.gov/pubmed?term=Zhang%20F%5BAuthor%5D&cauthor=true&cauthor_uid=21174522), [Dai Q](http://www.ncbi.nlm.nih.gov/pubmed?term=Dai%20Q%5BAuthor%5D&cauthor=true&cauthor_uid=21174522), [Cui H](http://www.ncbi.nlm.nih.gov/pubmed?term=Cui%20H%5BAuthor%5D&cauthor=true&cauthor_uid=21174522), [Wang DM](http://www.ncbi.nlm.nih.gov/pubmed?term=Wang%20DM%5BAuthor%5D&cauthor=true&cauthor_uid=21174522), [Yu J](http://www.ncbi.nlm.nih.gov/pubmed?term=Yu%20J%5BAuthor%5D&cauthor=true&cauthor_uid=21174522), [Hu S](http://www.ncbi.nlm.nih.gov/pubmed?term=Hu%20S%5BAuthor%5D&cauthor=true&cauthor_uid=21174522), [Lu S](http://www.ncbi.nlm.nih.gov/pubmed?term=Lu%20S%5BAuthor%5D&cauthor=true&cauthor_uid=21174522): Genetic linkage analyses and Cx50 mutation detection in a large multiplex Chinese family with hereditary nuclear cataract. *Ophthalmic Genet* 2011, 32:48-53. |
| 13 |  |  |  |  |  | AD | China | Nuclear, punctiform | [Wang L](http://www.ncbi.nlm.nih.gov/pubmed?term=Wang%20L%5BAuthor%5D&cauthor=true&cauthor_uid=21921990), [Luo Y](http://www.ncbi.nlm.nih.gov/pubmed?term=Luo%20Y%5BAuthor%5D&cauthor=true&cauthor_uid=21921990), [Wen W](http://www.ncbi.nlm.nih.gov/pubmed?term=Wen%20W%5BAuthor%5D&cauthor=true&cauthor_uid=21921990), [Zhang S](http://www.ncbi.nlm.nih.gov/pubmed?term=Zhang%20S%5BAuthor%5D&cauthor=true&cauthor_uid=21921990), [Lu Y](http://www.ncbi.nlm.nih.gov/pubmed?term=Lu%20Y%5BAuthor%5D&cauthor=true&cauthor_uid=21921990). Another evidence for a D47N mutation in GJA8 associated with autosomal dominant congenital cataract. *Mol Vis* 2011, 17:2380-2385. |
| 14 | c.142G>A | p.Glu48Lys | 0.04 | 1.000 | EC-1 | AD | Pakistan | Zonular nuclear pulverulent | [Berry V](http://www.ncbi.nlm.nih.gov/pubmed?term=Berry%20V%5BAuthor%5D&cauthor=true&cauthor_uid=10480374), [Mackay D](http://www.ncbi.nlm.nih.gov/pubmed?term=Mackay%20D%5BAuthor%5D&cauthor=true&cauthor_uid=10480374), [Khaliq S](http://www.ncbi.nlm.nih.gov/pubmed?term=Khaliq%20S%5BAuthor%5D&cauthor=true&cauthor_uid=10480374), [Francis PJ](http://www.ncbi.nlm.nih.gov/pubmed?term=Francis%20PJ%5BAuthor%5D&cauthor=true&cauthor_uid=10480374), [Hameed A](http://www.ncbi.nlm.nih.gov/pubmed?term=Hameed%20A%5BAuthor%5D&cauthor=true&cauthor_uid=10480374), Anwar K, [Mehdi SQ](http://www.ncbi.nlm.nih.gov/pubmed?term=Mehdi%20SQ%5BAuthor%5D&cauthor=true&cauthor_uid=10480374), [Newbold RJ](http://www.ncbi.nlm.nih.gov/pubmed?term=Newbold%20RJ%5BAuthor%5D&cauthor=true&cauthor_uid=10480374), [Ionides A](http://www.ncbi.nlm.nih.gov/pubmed?term=Ionides%20A%5BAuthor%5D&cauthor=true&cauthor_uid=10480374), [Shiels A](http://www.ncbi.nlm.nih.gov/pubmed?term=Shiels%20A%5BAuthor%5D&cauthor=true&cauthor_uid=10480374), [Moore T](http://www.ncbi.nlm.nih.gov/pubmed?term=Moore%20T%5BAuthor%5D&cauthor=true&cauthor_uid=10480374), [Bhattacharya SS](http://www.ncbi.nlm.nih.gov/pubmed?term=Bhattacharya%20SS%5BAuthor%5D&cauthor=true&cauthor_uid=10480374): Connexin 50 mutation in a family with congenital "zonular nuclear" pulverulent cataract of Pakistani origin. *Hum Genet* 1999, 105:168-170. |
| 15 | c.191T>G | p.Val64Gly | 0.00 | 0.999 | EC-1 | AD | China | Nuclear | [Zheng JQ](http://www.ncbi.nlm.nih.gov/pubmed?term=Zheng%20JQ%5BAuthor%5D&cauthor=true&cauthor_uid=15696487), [Ma ZW](http://www.ncbi.nlm.nih.gov/pubmed?term=Ma%20ZW%5BAuthor%5D&cauthor=true&cauthor_uid=15696487), [Sun HM](http://www.ncbi.nlm.nih.gov/pubmed?term=Sun%20HM%5BAuthor%5D&cauthor=true&cauthor_uid=15696487): [A heterozygous transversion of connexin 50 in a family with congenital nuclear cataract in the northeast of China]. *Zhonghua Yi Xue Yi Chuan Xue Za Zhi* 2005, 22:76-78. |
| 16 | c.218C>T | p.Ser73Phe | 0.00 | 1.000 | EC-1 | AD | Denmark | "Star-shaped" nuclear, polar | [Hansen L](http://www.ncbi.nlm.nih.gov/pubmed?term=Hansen%20L%5BAuthor%5D&cauthor=true&cauthor_uid=19182255), [Mikkelsen A](http://www.ncbi.nlm.nih.gov/pubmed?term=Mikkelsen%20A%5BAuthor%5D&cauthor=true&cauthor_uid=19182255), [Nürnberg P](http://www.ncbi.nlm.nih.gov/pubmed?term=N%C3%BCrnberg%20P%5BAuthor%5D&cauthor=true&cauthor_uid=19182255), [Nürnberg G](http://www.ncbi.nlm.nih.gov/pubmed?term=N%C3%BCrnberg%20G%5BAuthor%5D&cauthor=true&cauthor_uid=19182255), [Anjum I](http://www.ncbi.nlm.nih.gov/pubmed?term=Anjum%20I%5BAuthor%5D&cauthor=true&cauthor_uid=19182255), [Eiberg H](http://www.ncbi.nlm.nih.gov/pubmed?term=Eiberg%20H%5BAuthor%5D&cauthor=true&cauthor_uid=19182255), [Rosenberg T](http://www.ncbi.nlm.nih.gov/pubmed?term=Rosenberg%20T%5BAuthor%5D&cauthor=true&cauthor_uid=19182255): Comprehensive mutational screening in a cohort of Danish families with hereditary congenital cataract. [*Invest Ophthalmol Vis Sci*](http://www.ncbi.nlm.nih.gov/pubmed/19182255?ordinalpos=2&itool=EntrezSystem2.PEntrez.Pubmed.Pubmed_ResultsPanel.Pubmed_DefaultReportPanel.Pubmed_RVDocSum) 2009, 50:3291-3303. |
| 17 | c.235G>C | p.Val79Leu | 0.00 | 0.981 | TM-2 | AD | India | "Full moon" with Y-sutural opacities | [Vanita V](http://www.ncbi.nlm.nih.gov/pubmed?term=Vanita%20V%5BAuthor%5D&cauthor=true&cauthor_uid=17110920), [Hennies HC](http://www.ncbi.nlm.nih.gov/pubmed?term=Hennies%20HC%5BAuthor%5D&cauthor=true&cauthor_uid=17110920), [Singh D](http://www.ncbi.nlm.nih.gov/pubmed?term=Singh%20D%5BAuthor%5D&cauthor=true&cauthor_uid=17110920), [Nürnberg P](http://www.ncbi.nlm.nih.gov/pubmed?term=N%C3%BCrnberg%20P%5BAuthor%5D&cauthor=true&cauthor_uid=17110920), [Sperling K](http://www.ncbi.nlm.nih.gov/pubmed?term=Sperling%20K%5BAuthor%5D&cauthor=true&cauthor_uid=17110920), [Singh JR](http://www.ncbi.nlm.nih.gov/pubmed?term=Singh%20JR%5BAuthor%5D&cauthor=true&cauthor_uid=17110920): A novel mutation in GJA8 associated with autosomal dominant congenital cataract in a family of Indian origin. *Mol Vis* 2006, 12:1217-1222. |
| 18 | c.262C>T | p.Pro88Ser | 0.00 | 1.000 | TM-2 | AD | UK | Zonular pulverulent | Shiels A, Mackay D, Ionides A, Berry V, Moore A, Bhattacharya S: A missense mutation in the human connexin50 gene (*GJA8*) underlies autosomal dominant “zonular pulverulent” cataract on chromosome 1q. *Am J Hum Genet* 1998, 62:526-532. |
| 19 | c.262C>A | p.Pro88Thr | 0.000 | 0.999 | TM-2 | AD | China | Total | [Ge XL](http://www.ncbi.nlm.nih.gov/pubmed?term=Ge%20XL%5BAuthor%5D&cauthor=true&cauthor_uid=24535056), [Zhang Y](http://www.ncbi.nlm.nih.gov/pubmed?term=Zhang%20Y%5BAuthor%5D&cauthor=true&cauthor_uid=24535056), [Wu Y](http://www.ncbi.nlm.nih.gov/pubmed?term=Wu%20Y%5BAuthor%5D&cauthor=true&cauthor_uid=24535056), [Lv J](http://www.ncbi.nlm.nih.gov/pubmed?term=Lv%20J%5BAuthor%5D&cauthor=true&cauthor_uid=24535056), Zhang W, [Jin ZB](http://www.ncbi.nlm.nih.gov/pubmed?term=Jin%20ZB%5BAuthor%5D&cauthor=true&cauthor_uid=24535056), [Qu J](http://www.ncbi.nlm.nih.gov/pubmed?term=Qu%20J%5BAuthor%5D&cauthor=true&cauthor_uid=24535056), [Gu F](http://www.ncbi.nlm.nih.gov/pubmed?term=Gu%20F%5BAuthor%5D&cauthor=true&cauthor_uid=24535056): Identification of a novel GJA8 (Cx50) point mutation causes human dominant congenital cataracts. *Sci Rep* 2014, 4:4121. |
| 20 | c.263C>A | p.Pro88Gln | 0.00 | 1.000 | TM-2 | AD | UK | Lamellar pulverulent | [Arora A](http://www.ncbi.nlm.nih.gov/pubmed?term=Arora%20A%5BAuthor%5D&cauthor=true&cauthor_uid=16397066), [Minogue PJ](http://www.ncbi.nlm.nih.gov/pubmed?term=Minogue%20PJ%5BAuthor%5D&cauthor=true&cauthor_uid=16397066), [Liu X](http://www.ncbi.nlm.nih.gov/pubmed?term=Liu%20X%5BAuthor%5D&cauthor=true&cauthor_uid=16397066), [Reddy MA](http://www.ncbi.nlm.nih.gov/pubmed?term=Reddy%20MA%5BAuthor%5D&cauthor=true&cauthor_uid=16397066), [Ainsworth JR](http://www.ncbi.nlm.nih.gov/pubmed?term=Ainsworth%20JR%5BAuthor%5D&cauthor=true&cauthor_uid=16397066), [Bhattacharya SS](http://www.ncbi.nlm.nih.gov/pubmed?term=Bhattacharya%20SS%5BAuthor%5D&cauthor=true&cauthor_uid=16397066), [Webster AR](http://www.ncbi.nlm.nih.gov/pubmed?term=Webster%20AR%5BAuthor%5D&cauthor=true&cauthor_uid=16397066), [Hunt DM](http://www.ncbi.nlm.nih.gov/pubmed?term=Hunt%20DM%5BAuthor%5D&cauthor=true&cauthor_uid=16397066), [Ebihara L](http://www.ncbi.nlm.nih.gov/pubmed?term=Ebihara%20L%5BAuthor%5D&cauthor=true&cauthor_uid=16397066), [Moore AT](http://www.ncbi.nlm.nih.gov/pubmed?term=Moore%20AT%5BAuthor%5D&cauthor=true&cauthor_uid=16397066), Beyer EC, [Berthoud VM](http://www.ncbi.nlm.nih.gov/pubmed?term=Berthoud%20VM%5BAuthor%5D&cauthor=true&cauthor_uid=16397066): A novel GJA8 mutation is associated with autosomal dominant lamellar pulverulent cataract: further evidence for gap junction dysfunction in human cataract. *J Med Gene*t 2006, 43:e2. |
| 21 |  |  |  |  |  | AD | India | Balloon-like, sutural | [Vanita V](http://www.ncbi.nlm.nih.gov/pubmed?term=Vanita%20V%5BAuthor%5D&cauthor=true&cauthor_uid=18587493), [Singh JR](http://www.ncbi.nlm.nih.gov/pubmed?term=Singh%20JR%5BAuthor%5D&cauthor=true&cauthor_uid=18587493), Singh D, [Varon R](http://www.ncbi.nlm.nih.gov/pubmed?term=Varon%20R%5BAuthor%5D&cauthor=true&cauthor_uid=18587493), [Sperling K](http://www.ncbi.nlm.nih.gov/pubmed?term=Sperling%20K%5BAuthor%5D&cauthor=true&cauthor_uid=18587493): A mutation in GJA8 (p.P88Q) is associated with "balloon-like" cataract with Y-sutural opacities in a family of Indian origin. *Mol Vis* 2008, 14:1171-1175. |
| 22 | c.293A>C | p.His98Pro | 0.00 | 1.000 | TM-2 | AD | USA |  | This study |
| 23 | c.566C>T | p.Pro189Leu | 0.00 | 1.000 | EC-2 | AD | Denmark | "Star shaped" nuclear (microcornea) | Hansen L, Yao W, [Eiberg H](http://www.ncbi.nlm.nih.gov/pubmed?term=Eiberg%20H%5BAuthor%5D&cauthor=true&cauthor_uid=17724170), Kjaer KW, Baggesen K, Hejtmancik JF, Rosenberg T: Genetic heterogeneity in microcornea-cataract: five novel mutations in CRYAA, CRYGD, and GJA8. *Invest Ophthalmol Vis Sci* 2007, 48:3937-3944. |
| 24 | c.586G>A | p.V196M | 0.00 | 1.000 | EC-2 | AR | India | Total | [Ponnam SP](http://www.ncbi.nlm.nih.gov/pubmed?term=Ponnam%20SP%5BAuthor%5D&cauthor=true&cauthor_uid=23734083), [Ramesha K](http://www.ncbi.nlm.nih.gov/pubmed?term=Ramesha%20K%5BAuthor%5D&cauthor=true&cauthor_uid=23734083), [Matalia J](http://www.ncbi.nlm.nih.gov/pubmed?term=Matalia%20J%5BAuthor%5D&cauthor=true&cauthor_uid=23734083), [Tejwani S](http://www.ncbi.nlm.nih.gov/pubmed?term=Tejwani%20S%5BAuthor%5D&cauthor=true&cauthor_uid=23734083), Ramamurthy B, Kannabiran C: Mutational screening of Indian families with hereditary congenital cataract. *Mol Vis* 2013, 19:1141-1148. |
| 25 | c.592C>T | p.Arg198Trp | 0.00 | 1.000 | EC-2 | AD | China | (microcornea) | [Hu S](http://www.ncbi.nlm.nih.gov/pubmed?term=Hu%20S%5BAuthor%5D&cauthor=true&cauthor_uid=20806042), [Wang B](http://www.ncbi.nlm.nih.gov/pubmed?term=Wang%20B%5BAuthor%5D&cauthor=true&cauthor_uid=20806042), [Zhou Z](http://www.ncbi.nlm.nih.gov/pubmed?term=Zhou%20Z%5BAuthor%5D&cauthor=true&cauthor_uid=20806042), [Zhou G](http://www.ncbi.nlm.nih.gov/pubmed?term=Zhou%20G%5BAuthor%5D&cauthor=true&cauthor_uid=20806042), Wang J, [Ma X](http://www.ncbi.nlm.nih.gov/pubmed?term=Ma%20X%5BAuthor%5D&cauthor=true&cauthor_uid=20806042), [Qi Y](http://www.ncbi.nlm.nih.gov/pubmed?term=Qi%20Y%5BAuthor%5D&cauthor=true&cauthor_uid=20806042): A novel mutation in GJA8 causing congenital cataract-microcornea syndrome in a Chinese pedigree. *Mol Vis* 2010, 16:1585-1592. |
| 26 | c.593G>A | p.Arg198Gln | 0.00 | 1.000 | EC-2 | AD | India | Posterior subcapsular (microcornea, mild myopia) | Devi RR, [Vijayalakshmi P](http://www.ncbi.nlm.nih.gov/pubmed?term=Vijayalakshmi%20P%5BAuthor%5D&cauthor=true&cauthor_uid=16604058): Novel mutations in GJA8 associated with autosomal dominant congenital cataract and microcornea. *Mol Vis* 2006, 12:190-195. |
| 27 |  |  |  |  |  |  | Australia | Nuclear (microcornea) | Prokudin I, [Simons C](http://www.ncbi.nlm.nih.gov/pubmed?term=Simons%20C%5BAuthor%5D&cauthor=true&cauthor_uid=24281366), [Grigg JR](http://www.ncbi.nlm.nih.gov/pubmed?term=Grigg%20JR%5BAuthor%5D&cauthor=true&cauthor_uid=24281366), [Storen R](http://www.ncbi.nlm.nih.gov/pubmed?term=Storen%20R%5BAuthor%5D&cauthor=true&cauthor_uid=24281366), [Kumar V](http://www.ncbi.nlm.nih.gov/pubmed?term=Kumar%20V%5BAuthor%5D&cauthor=true&cauthor_uid=24281366), [Phua ZY](http://www.ncbi.nlm.nih.gov/pubmed?term=Phua%20ZY%5BAuthor%5D&cauthor=true&cauthor_uid=24281366), [Smith J](http://www.ncbi.nlm.nih.gov/pubmed?term=Smith%20J%5BAuthor%5D&cauthor=true&cauthor_uid=24281366), [Flaherty M](http://www.ncbi.nlm.nih.gov/pubmed?term=Flaherty%20M%5BAuthor%5D&cauthor=true&cauthor_uid=24281366), [Davila S](http://www.ncbi.nlm.nih.gov/pubmed?term=Davila%20S%5BAuthor%5D&cauthor=true&cauthor_uid=24281366), [Jamieson RV](http://www.ncbi.nlm.nih.gov/pubmed?term=Jamieson%20RV%5BAuthor%5D&cauthor=true&cauthor_uid=24281366): Exome sequencing in developmental eye disease leads to identification of causal variants in GJA8, CRYGC, PAX6 and CYP1B1. *Eur J Hum Genet* 2014, 22:907-915. |
| 28 | c.595C>T | p.Pro199Ser | 0.00 | 1.00 | EC-2 | AD | India |  | [Ponnam SP](http://www.ncbi.nlm.nih.gov/pubmed?term=Ponnam%20SP%5BAuthor%5D&cauthor=true&cauthor_uid=23734083), [Ramesha K](http://www.ncbi.nlm.nih.gov/pubmed?term=Ramesha%20K%5BAuthor%5D&cauthor=true&cauthor_uid=23734083), [Matalia J](http://www.ncbi.nlm.nih.gov/pubmed?term=Matalia%20J%5BAuthor%5D&cauthor=true&cauthor_uid=23734083), [Tejwani S](http://www.ncbi.nlm.nih.gov/pubmed?term=Tejwani%20S%5BAuthor%5D&cauthor=true&cauthor_uid=23734083), Ramamurthy B, Kannabiran C: Mutational screening of Indian families with hereditary congenital cataract. *Mol Vis* 2013, 19:1141-1148. |
| 29 | c.601G>A | p.Glu201Lys | 0.00 | 0.999 | EC-2 | AD | China | Perinuclear | [Su D](http://www.ncbi.nlm.nih.gov/pubmed?term=Su%20D%5BAuthor%5D&cauthor=true&cauthor_uid=23555834), Yang Z, [Li Q](http://www.ncbi.nlm.nih.gov/pubmed?term=Li%20Q%5BAuthor%5D&cauthor=true&cauthor_uid=23555834), [Guan L](http://www.ncbi.nlm.nih.gov/pubmed?term=Guan%20L%5BAuthor%5D&cauthor=true&cauthor_uid=23555834), [Zhang H](http://www.ncbi.nlm.nih.gov/pubmed?term=Zhang%20H%5BAuthor%5D&cauthor=true&cauthor_uid=23555834), [E D](http://www.ncbi.nlm.nih.gov/pubmed?term=E%20D%5BAuthor%5D&cauthor=true&cauthor_uid=23555834), [Zhang L](http://www.ncbi.nlm.nih.gov/pubmed?term=Zhang%20L%5BAuthor%5D&cauthor=true&cauthor_uid=23555834), [Zhu S](http://www.ncbi.nlm.nih.gov/pubmed?term=Zhu%20S%5BAuthor%5D&cauthor=true&cauthor_uid=23555834), [Ma X](http://www.ncbi.nlm.nih.gov/pubmed?term=Ma%20X%5BAuthor%5D&cauthor=true&cauthor_uid=23555834): Identification and functional analysis of GJA8 mutation in a Chinese family with autosomal dominant perinuclear cataracts. *PLoS One* 2013, 8(3):e59926. |
| 30 | c.608insA | p.Thr203AsnfsX47 |  |  | EC-2 | AR | India | Total (nystagmus) | [Ponnam SP](http://www.ncbi.nlm.nih.gov/pubmed?term=Ponnam%20SP%5BAuthor%5D&cauthor=true&cauthor_uid=17601931), [Ramesha K](http://www.ncbi.nlm.nih.gov/pubmed?term=Ramesha%20K%5BAuthor%5D&cauthor=true&cauthor_uid=17601931), [Tejwani S](http://www.ncbi.nlm.nih.gov/pubmed?term=Tejwani%20S%5BAuthor%5D&cauthor=true&cauthor_uid=17601931), [Ramamurthy B](http://www.ncbi.nlm.nih.gov/pubmed?term=Ramamurthy%20B%5BAuthor%5D&cauthor=true&cauthor_uid=17601931), [Kannabiran C](http://www.ncbi.nlm.nih.gov/pubmed?term=Kannabiran%20C%5BAuthor%5D&cauthor=true&cauthor_uid=17601931): Mutation of the gap junction protein alpha 8 (GJA8) gene causes autosomal recessive cataract. *J Med Genet* 2007, 44:e85. |
| 31 | c.741T>G | p.Ile247Met | 0.08 | 0.468 | C-Term | AD | Russia | Zonular pulverulent | [Polyakov AV](http://www.ncbi.nlm.nih.gov/pubmed?term=Polyakov%20AV%5BAuthor%5D&cauthor=true&cauthor_uid=11846744), [Shagina IA](http://www.ncbi.nlm.nih.gov/pubmed?term=Shagina%20IA%5BAuthor%5D&cauthor=true&cauthor_uid=11846744), [Khlebnikova OV](http://www.ncbi.nlm.nih.gov/pubmed?term=Khlebnikova%20OV%5BAuthor%5D&cauthor=true&cauthor_uid=11846744), [Evgrafov OV](http://www.ncbi.nlm.nih.gov/pubmed?term=Evgrafov%20OV%5BAuthor%5D&cauthor=true&cauthor_uid=11846744): Mutation in the connexin 50 gene (GJA8) in a Russian family with zonular pulverulent cataract. *Clin Genet* 2001, 60:476-478. |
| 32 |  |  |  |  |  |  | Germany | Congenital, bilateral, white, diffuse | [Graw J](http://www.ncbi.nlm.nih.gov/pubmed?term=Graw%20J%5BAuthor%5D&cauthor=true&cauthor_uid=19756179), [Schmidt W](http://www.ncbi.nlm.nih.gov/pubmed?term=Schmidt%20W%5BAuthor%5D&cauthor=true&cauthor_uid=19756179), [Minogue PJ](http://www.ncbi.nlm.nih.gov/pubmed?term=Minogue%20PJ%5BAuthor%5D&cauthor=true&cauthor_uid=19756179), Rodriguez J, [Tong JJ](http://www.ncbi.nlm.nih.gov/pubmed?term=Tong%20JJ%5BAuthor%5D&cauthor=true&cauthor_uid=19756179), [Klopp N](http://www.ncbi.nlm.nih.gov/pubmed?term=Klopp%20N%5BAuthor%5D&cauthor=true&cauthor_uid=19756179), [Illig T](http://www.ncbi.nlm.nih.gov/pubmed?term=Illig%20T%5BAuthor%5D&cauthor=true&cauthor_uid=19756179), [Ebihara L](http://www.ncbi.nlm.nih.gov/pubmed?term=Ebihara%20L%5BAuthor%5D&cauthor=true&cauthor_uid=19756179), [Berthoud VM](http://www.ncbi.nlm.nih.gov/pubmed?term=Berthoud%20VM%5BAuthor%5D&cauthor=true&cauthor_uid=19756179), [Beyer EC](http://www.ncbi.nlm.nih.gov/pubmed?term=Beyer%20EC%5BAuthor%5D&cauthor=true&cauthor_uid=19756179): The GJA8 allele encoding CX50I247M is a rare polymorphism, not a cataract-causing mutation. *Mol Vis* 2009, 15:1881-1885. |
| 33 | c.767insG | p.Ala256GLyfsX124 |  |  | C-Term | AR | Germany | Triangular | [Schmidt W](http://www.ncbi.nlm.nih.gov/pubmed?term=Schmidt%20W%5BAuthor%5D&cauthor=true&cauthor_uid=18483562), [Klopp N](http://www.ncbi.nlm.nih.gov/pubmed?term=Klopp%20N%5BAuthor%5D&cauthor=true&cauthor_uid=18483562), [Illig T](http://www.ncbi.nlm.nih.gov/pubmed?term=Illig%20T%5BAuthor%5D&cauthor=true&cauthor_uid=18483562), [Graw J](http://www.ncbi.nlm.nih.gov/pubmed?term=Graw%20J%5BAuthor%5D&cauthor=true&cauthor_uid=18483562): A novel GJA8 mutation causing a recessive triangular cataract. *Mol Vis* 2008, 14:851-856. |
| 34 | c.773C>T | p.Ser258Phe | 0.00 | 0.991 | C-Term | AD | China | Congenital nuclear | [Gao X](http://www.ncbi.nlm.nih.gov/pubmed?term=Gao%20X%5BAuthor%5D&cauthor=true&cauthor_uid=20597646), [Cheng J](http://www.ncbi.nlm.nih.gov/pubmed?term=Cheng%20J%5BAuthor%5D&cauthor=true&cauthor_uid=20597646), [Lu C](http://www.ncbi.nlm.nih.gov/pubmed?term=Lu%20C%5BAuthor%5D&cauthor=true&cauthor_uid=20597646), [Li X](http://www.ncbi.nlm.nih.gov/pubmed?term=Li%20X%5BAuthor%5D&cauthor=true&cauthor_uid=20597646), [Li F](http://www.ncbi.nlm.nih.gov/pubmed?term=Li%20F%5BAuthor%5D&cauthor=true&cauthor_uid=20597646), [Liu C](http://www.ncbi.nlm.nih.gov/pubmed?term=Liu%20C%5BAuthor%5D&cauthor=true&cauthor_uid=20597646), [Zhang M](http://www.ncbi.nlm.nih.gov/pubmed?term=Zhang%20M%5BAuthor%5D&cauthor=true&cauthor_uid=20597646), [Zhu S](http://www.ncbi.nlm.nih.gov/pubmed?term=Zhu%20S%5BAuthor%5D&cauthor=true&cauthor_uid=20597646), [Ma X](http://www.ncbi.nlm.nih.gov/pubmed?term=Ma%20X%5BAuthor%5D&cauthor=true&cauthor_uid=20597646): A novel mutation in the connexin 50 gene (GJA8) associated with autosomal dominant congenital nuclear cataract in a Chinese family. *Curr Eye Res* 2010, 35:597-604.  Chen J-H, Qiu J, Chen H, Pang CP, Zhang M: Rapid and cost-effective molecular diagnosis using exome sequencing of one proband with autosomal dominant congenital cataract. Eye 2014, Oct 10. doi: 10.1038/eye.2014.158. [Epub ahead of print]. |
| 35 | c.836C>A | p.Ser259Tyr | 0.00 | 0.998 | C-Term | AD | Denmark |  | [Hansen L](http://www.ncbi.nlm.nih.gov/pubmed?term=Hansen%20L%5BAuthor%5D&cauthor=true&cauthor_uid=19182255), [Mikkelsen A](http://www.ncbi.nlm.nih.gov/pubmed?term=Mikkelsen%20A%5BAuthor%5D&cauthor=true&cauthor_uid=19182255), [Nürnberg P](http://www.ncbi.nlm.nih.gov/pubmed?term=N%C3%BCrnberg%20P%5BAuthor%5D&cauthor=true&cauthor_uid=19182255), [Nürnberg G](http://www.ncbi.nlm.nih.gov/pubmed?term=N%C3%BCrnberg%20G%5BAuthor%5D&cauthor=true&cauthor_uid=19182255), [Anjum I](http://www.ncbi.nlm.nih.gov/pubmed?term=Anjum%20I%5BAuthor%5D&cauthor=true&cauthor_uid=19182255), [Eiberg H](http://www.ncbi.nlm.nih.gov/pubmed?term=Eiberg%20H%5BAuthor%5D&cauthor=true&cauthor_uid=19182255), [Rosenberg T](http://www.ncbi.nlm.nih.gov/pubmed?term=Rosenberg%20T%5BAuthor%5D&cauthor=true&cauthor_uid=19182255): Comprehensive mutational screening in a cohort of Danish families with hereditary congenital cataract. *Invest Ophthalmol Vis Sci* 2009, 50:3291-3303. |
| 36 | c.827C>T | p.Ser276Phe | 0.08 | 0.068 | C-Term | AD | China | Nuclear pulverulent | [Yan M](http://www.ncbi.nlm.nih.gov/pubmed?term=Yan%20M%5BAuthor%5D&cauthor=true&cauthor_uid=18334966), [Xiong C](http://www.ncbi.nlm.nih.gov/pubmed?term=Xiong%20C%5BAuthor%5D&cauthor=true&cauthor_uid=18334966), [Ye SQ](http://www.ncbi.nlm.nih.gov/pubmed?term=Ye%20SQ%5BAuthor%5D&cauthor=true&cauthor_uid=18334966), [Chen Y](http://www.ncbi.nlm.nih.gov/pubmed?term=Chen%20Y%5BAuthor%5D&cauthor=true&cauthor_uid=18334966), Ke M, [Zheng F](http://www.ncbi.nlm.nih.gov/pubmed?term=Zheng%20F%5BAuthor%5D&cauthor=true&cauthor_uid=18334966), [Zhou X](http://www.ncbi.nlm.nih.gov/pubmed?term=Zhou%20X%5BAuthor%5D&cauthor=true&cauthor_uid=18334966): A novel connexin 50 (GJA8) mutation in a Chinese family with a dominant congenital pulverulent nuclear cataract. *Mol Vis* 2008, 14:418-424. |
| 37 | c.842T>C | p.Leu281Ser | 0.00 | 0.999 | C-Term | AD | India | Lamellar/zonular (nystagmus) | [Kumar M](http://www.ncbi.nlm.nih.gov/pubmed?term=Kumar%20M%5BAuthor%5D&cauthor=true&cauthor_uid=21423869), [Agarwal T](http://www.ncbi.nlm.nih.gov/pubmed?term=Agarwal%20T%5BAuthor%5D&cauthor=true&cauthor_uid=21423869), [Khokhar S](http://www.ncbi.nlm.nih.gov/pubmed?term=Khokhar%20S%5BAuthor%5D&cauthor=true&cauthor_uid=21423869), Kumar M, [Kaur P](http://www.ncbi.nlm.nih.gov/pubmed?term=Kaur%20P%5BAuthor%5D&cauthor=true&cauthor_uid=21423869), [Roy TS](http://www.ncbi.nlm.nih.gov/pubmed?term=Roy%20TS%5BAuthor%5D&cauthor=true&cauthor_uid=21423869), [Dada R](http://www.ncbi.nlm.nih.gov/pubmed?term=Dada%20R%5BAuthor%5D&cauthor=true&cauthor_uid=21423869): Mutation screening and genotype phenotype correlation of α-crystallin, γ-crystallin and GJA8 gene in congenital cataract. *Mol Vis* 2011, 17:693-707. |
