# Supplementary material for: Exome sequencing identifies novel and recurrent mutations in GJA8 and CRYGD associated with inherited cataract
Source: Hum Genomics. 2014 Nov 18;8(1):19. doi: 10.1186/s40246-014-0019-6 (PMC4240822; doi:10.1186/s40246-014-0019-6)
Supplement: Additional file 6: Table S6. — PCR primers used for Sanger sequencing of mutations found in CRYGD and GJA8. Each primer used was tailed with M13 sequences to aid in Sanger sequencing. The forward primers were tagged with ‘tgtaaaacgacggccagt’ and the reverse primers with ‘caggaaacagctatgacc’. [file 40246_2014_19_MOESM6_ESM.docx]

**Additional file 6. Table S6.** PCR primers used for Sanger sequencing of mutations found in *CRYGD* and *GJA8*.

| Primer | Location | Strand | Sequence (5´ - 3´) |
| --- | --- | --- | --- |
| CRYGD-2F | Exon 2 | Sense | GCCCGAGTAGTTGGGCTG3 |
| CRYGD-2R | Exon 2 | Antisense | TGAAATAGCTGAAGCTCC3 |
| GJA8-2F | Exon 2 | Sense | CGCTGTGTGCACATTGACCG3 |
| GJA8-2R | Exon 2 | Antisense | TGGCAGATGTAGGTCCTCAGCA |

Forward PCR primers were tagged with an M13 sequencing primer (5´-tgtaaaacgacggccagt)

Reverse PCR primers were tagged with an M13 sequencing primer (5´-caggaaacagctatgacc)
